# Highly Recurrent Multinucleotide Mutations in SARS-CoV-2

**DOI:** 10.1093/molbev/msaf272

**Published:** 2025-10-24

**Authors:** Nicola De Maio, Olivier Anoufa, Kyle Smith, Yatish Turakhia, Nick Goldman

**Affiliations:** European Molecular Biology Laboratory-European Bioinformatics Institute, EMBL-EBI, Wellcome Genome Campus, Hinxton, Cambridgeshire CB10 1SD, UK; European Molecular Biology Laboratory-European Bioinformatics Institute, EMBL-EBI, Wellcome Genome Campus, Hinxton, Cambridgeshire CB10 1SD, UK; Faculty of Sciences and Technologies, Université de Lille, Centrale Lille, IMT Nord Europe, Lille 59000, France; Department of Electrical and Computer Engineering, University of California San Diego, San Diego, CA 92093, USA; Department of Electrical and Computer Engineering, University of California San Diego, San Diego, CA 92093, USA; European Molecular Biology Laboratory-European Bioinformatics Institute, EMBL-EBI, Wellcome Genome Campus, Hinxton, Cambridgeshire CB10 1SD, UK

**Keywords:** SARS-CoV-2, pandemic-scale phylogenetics, multinucleotide mutations, complex mutations, recurrent mutations

## Abstract

Multinucleotide mutations simultaneously replace multiple nucleotides. They are a significant contributor to evolution and disease, as well as to misdiagnosis, misannotation and other biases in genome data analysis. Multinucleotide mutations are generally thought to be rare and random events. However, by processing over 2 million publicly shared genomes, we show that certain multinucleotide mutations are highly recurrent in SARS-CoV-2: they repeatedly and consistently modify the same multiple nucleotides at the same genome position in the same way. The most frequent of these multinucleotide mutations have independently occurred hundreds of times across all SARS-CoV-2 lineages. We find evidence that the vast majority of these recurrent multinucleotide mutations (14 out of 15, corresponding to 97.6% of all individual occurrences) are linked to transcription regulatory sequences. We propose a mechanism that can explain them through template switching as part of the natural transcription process of the virus. This previously unknown mutational pattern increases our understanding of the evolution of SARS-CoV-2 and potentially many other nidoviruses. It also has important consequences for computational evolutionary biology: we show that for example recurrent multinucleotide mutations cause approximately 12% of false positives during inference of recombination in SARS-CoV-2.

## Introduction

Single-nucleotide substitutions are the most frequent type of genetic mutation (Kloosterman et al. 2015), and are often the only ones considered in genome data analyses such as in population genetics (Hamilton 2021) and phylogenetics (Yang and Rannala 2012). However, other types of mutations also significantly contribute to genome evolution. Among these, multinucleotide mutations (MNMs), mutational events simultaneously replacing multiple nearby nucleotides, are some of the most debated (Averof et al. 2000; Kondrashov 2003; Smith et al. 2003; Kosiol et al. 2007).

Early evidence for MNMs came from multiple nucleotide substitutions (mnSs: multiple nearby genetic differences observed between species), see Golding and Glickman (1985), Averof et al. (2000), Whelan and Goldman (2004) and Kosiol et al. (2007). Although the terms mnS and MNM are often used interchangeably, we define mnSs as nucleotide substitutions inferred on the same phylogenetic tree branch. An mnS could be the result of distinct sequential single-nucleotide mutation events, or of a single mutation event. On the other hand, we use the term MNM to refer to mutation events causing the instantaneous replacement of multiple nearby nucleotides. MNMs typically cause mnSs, but not all mnSs are attributable to MNMs, since successive single-nucleotide mutations occurring on the same phylogenetic branch also cause mnSs.

Researchers have debated what proportion of mnSs (and specifically of mnSs comprising nearby nucleotide substitutions) is caused by MNMs, and what proportion might be caused by other factors such as selection (Bazykin et al. 2004; Belinky et al. 2019) and variation in substitution rates (Kondrashov 2003; Smith et al. 2003). However, multiple lines of evidence including trio sequencing and mutation accumulation lines have confirmed the existence of MNMs in different species, and have estimated their prevalence to be between 0.4% and 10% of total nucleotide mutations (Hampsey et al. 1988; Nakazawa et al. 1994; Hodgkinson and Eyre-Walker 2010; Schrider et al. 2011; Chen et al. 2014; Assaf et al. 2017; Wang et al. 2020). Causative mechanisms of MNMs include short-range template switches (Löytynoja and Goldman 2017; Walker et al. 2021; Löytynoja 2022) and ultraviolet light DNA damage (Nakazawa et al. 1994). MNMs have been reported to be a significant source of disease in humans (Kaplanis et al. 2019; Wang et al. 2022) and other animals (Degalez et al. 2021).

In computational biology, typically only single-nucleotide events are considered, causing problems like misannotation, and consequently misdiagnosis (Wakeling et al. 2019; Srinivasan et al. 2021). MNMs have also been shown to cause biases and false positives in positive selection inference (Venkat et al. 2018; Dunn et al. 2019; Lucaci et al. 2021, 2023). For this reason, several authors have proposed the inclusion of MNMs in phylogenetic substitution models (Kosiol et al. 2007; De Maio et al. 2013; Dunn et al. 2019; Lucaci et al. 2021, 2023).

The millions of SARS-COV-2 genomes shared publicly during the COVID-19 pandemic provide the unprecedented opportunity to study the virus’s evolution with extraordinary power and detail (De Maio et al. 2021; Markov et al. 2023; Sanderson et al. 2023). Here, we use a highly curated dataset of >2 million SARS-CoV-2 genomes (De Maio et al. 2024) to investigate MNMs. We take advantage of the low level of divergence, high density of sampling and large number of genomes in this dataset to reconstruct the mutational history of the virus with near certainty and to investigate site-specific mutational patterns with great detail and precision (De Maio et al. 2021, 2024). Focusing on highly recurrent mnSs, we identify some as attributable to recurrent sequence artifacts but conclude that the majority (15 of the 19 most frequently occurring) are caused by recurrent MNMs. Some of these have occurred hundreds of times as identical but distinct mutation events during SARS-CoV-2 evolution. We find strong evidence that most recurrent MNMs (13 out of 15, responsible for 91% of individual occurrences of MNM we have focused on) are caused by interrupted template switching, and we show that highly recurrent MNMs cause artifacts in the inference of recombination.

## Results

We investigated a mutation-annotated tree (a phylogenetic tree enriched with the information of which mutation was estimated to have occurred on which branch) inferred from a public dataset of 2,072,111 SARS-CoV-2 genomes (De Maio et al. 2024; Hunt et al. 2024, see Methods section). We define an mnS to be any group of nucleotide substitutions occurring on the same branch, and do not impose any constraint based on the location along the genome of the substitutions involved. This means that we also keep track of groups of substitutions far from each other along the genome, allowing for the possibility of mechanisms creating simultaneous distant substitutions. Defining recurrence as the number of times a substitution occurs along the phylogenetic tree (not the number of genomes containing the substitutions), we list the most highly recurrent mnSs in Table 1. The most recurrent mnSs found were the mutation C21304A-G21305A, inferred to have arisen 736 times: 452 times on its own (MNM1a, observed in 5,330 genomes) and 284 separate times as part of the larger MNM1 which also contains C21302T; and A28877T-G28878C (MNM2), which was inferred to have occurred 480 times and was observed in 14,714 genomes.

**Table 1.** Highly recurrent mnSs.

ID	mnS	Number of occurrences	Individual occurrences	Proportion of singletons	Number of observed genomes	Observed genomes per occurrence	*P*-value	Occurrences in simulations	Proposed mechanism	Protein affected
MNM1	C21302T	284	219	0.59	4197	14.8	$<10^{-300}$	0 (0–0)	plausible interrupted	ORF1b, nsp16: P2612L
	-C21304A		369				$\left(<10^{-300}\right)$		template switching	ORF1b, nsp16: R2613N
	-G21305A		290							
> MNM1a	C21304A	452	369	0.63	5330	11.8	$<10^{-300}$	1 (0–5)	plausible interrupted	ORF1b, nsp16: R2613N
	-G21305A		290				$\left(<10^{-300}\right)$		template switching	
MNM2	A28877T	480	50	0.62	14714	30.7	$<10^{-300}$	0 (0–2)	interrupted	N: synonymous
	-G28878C		18				$\left(<10^{-300}\right)$		template switching	(ORF9c: V49L)*
MNM3	G27382C	253	135	0.66	141947	561	$<10^{-300}$	0 (0–0)	interrupted	ORF6: D61L
	-A27383T		75				$\left(<10^{-300}\right)$		template switching	
	-T27384C		1724							
MNM4	T26491C	160	21	0.72	351	2.2	$<10^{-300}$	0 (0–0)	plausible interrupted	(intergenic)
	-A26492T		36				$\left(<10^{-300}\right)$		template switching	
	-T26497C		137							
MNM5	G27758A	126	43	0.64	312	2.5	$<10^{-300}$	0 (0–1)	plausible interrupted	ORF7a: synonymous
	-T27760A		60				$\left(<10^{-300}\right)$		template switching	ORF7b: M1I, I2N
MNM6	C25162A	116	21	0.60	624	5.4	$<10^{-300}$	0 (0–1)	plausible interrupted	S: Q1201K
	-C25163A		106				$\left(<10^{-300}\right)$		template switching	
MNM7	T27875C	49	33	0.55	1204	24.6	$6\cdot 10^{-202}$	0 (0–0)	interrupted	ORF7b: frameshift
	-C27881T		470				$\left(4\cdot 10^{-192}\right)$		template switching	
	-G27882C		25							
	-C27883T		198							
> MNM7a	C27881T	61	470	0.64	442	7.2	$4\cdot 10^{-216}$	0 (0–0)	interrupted	ORF7b: frameshift
	-G27882C		25				$\left(3\cdot 10^{-206}\right)$		template switching	
	-C27883T		198							
MNM8	T21294A	89	18	0.58	444	5.0	$<10^{-300}$	0 (0–0)	plausible interrupted	ORF1b, nsp16: G2610N
	-G21295A		49				$\left(<10^{-300}\right)$		template switching	
	-G21296A		58							
**mnS1**	**A507T**	89	7	0.82	166	1.9	$<10^{-300}$	0 (0–0)	**sequencing error**	
	**-T508C**		69				$\left(<10^{-300}\right)$		**due to incomplete**	
	**-G509A**		73						**read trimming**	
MNM9	A27038T	87	7	0.83	195	2.2	$<10^{-300}$	0 (0–0)	interrupted	M: S173K
	-T27039A		17				$\left(<10^{-300}\right)$		template switching	
	-C27040A		10							
MNM10	A21550C	68	14	0.72	202	3.0	$<10^{-300}$	0 (0–0)	interrupted	ORF1b, nsp16: N2694 **-**
	-A21551T		43				$\left(5\cdot 10^{-298}\right)$		template switching	ORF1b, nsp16: N2695L
**mnS2**	**T21994C**	64	55	0.67	103	1.6	$7\cdot 10^{-247}$	0 (0–1)	**sequencing errors**	
	**-T21995C**		139				$\left(6\cdot 10^{-237}\right)$		**due to deletion**	
MNM11	C13423A	62	16	0.69	157	2.5	$8\cdot 10^{-253}$	0 (0–1)	plausible interrupted	ORF1a, nsp10: R4387S
	-C13424A		165				$\left(6\cdot 10^{-243}\right)$		template switching	
MNM12	A4576T	60	22	0.62	2330	38.8	$5\cdot 10^{-213}$	0 (0–1)	plausible interrupted	ORF1a, nsp3: synonymous
	-T4579A		533				$\left(4\cdot 10^{-203}\right)$		template switching	
**mnS3**	**T28881A**	59	19	0.58	848359	14379	$2\cdot 10^{-275}$	0 (0–0)	**sequence errors due**	
	**-G28882A**		42				$\left(2\cdot 10^{-265}\right)$		**to reference bias**	
	**-G28883C**		19							
MNM13	A20284T	57	1	0.61	138	2.4	$9\cdot 10^{-291}$	0 (0–0)	unknown MNM	ORF1b, nsp15: I2273S
	-T20285C		7				$\left(8\cdot 10^{-281}\right)$			
**mnS4**	**G11083T**	53	11206	0.81	826	15.6	$9\cdot 10^{-11}$	66 (52–88)	**successive single-**	
	**-C21575T**		6913				(0.73)		**nt substitutions**	

Genome positions of all substitutions are given with respect to the SARS-CoV-2 Wuhan-Hu-1 reference genome MN908947.3 (Wu et al. 2020). Those unlikely to be caused by recurrent MNMs are highlighted in bold.

Number of occurrences: number of times the mnS is inferred to have arisen. Occurrences of larger mnSs are not considered when counting occurrences of smaller nested mnSs (indicated by >). For example, occurrences of MNM1 (C21302T-C21304A-G21305A) do not contribute to the count shown for MNM1a (C21304A-G21305A), although the latter MNM is contained in the former.

Individual occurrences: numbers of inferred substitutions for each single-nucleotide substitution composing the mnS, without counting occurrences of the mnS itself; for example, the first row states that 219 C21302T substitutions were inferred.

Proportion of singletons: proportions of mnS events with exactly one descendant.

Number of observed genomes: number of genomes in which the mutated version of the mnS is observed.

Observed genomes per occurrence: value in column 5 divided by the one in column 2, that is, the mean number of descendants of an mnS event.

*P*-value: significance of the hypergeometric test of the correlation between the individual substitutions composing the mnS (in parenthesis: Bonferroni-corrected values).

Occurrences in simulations: median number of times the mnS occurred across 100 simulation replicates (in parentheses: minimum and maximum numbers across replicates).

Proposed mechanism: proposed cause of the recurrent mnS (in bold when cause is not an MNM).

Protein affected: protein affected by the MNM and type of change (omitted for non-MNM mnSs). *ORF9c is in parentheses since it is not clear if it is functionally expressed (Finkel et al. 2021).

**Fig. 1.**
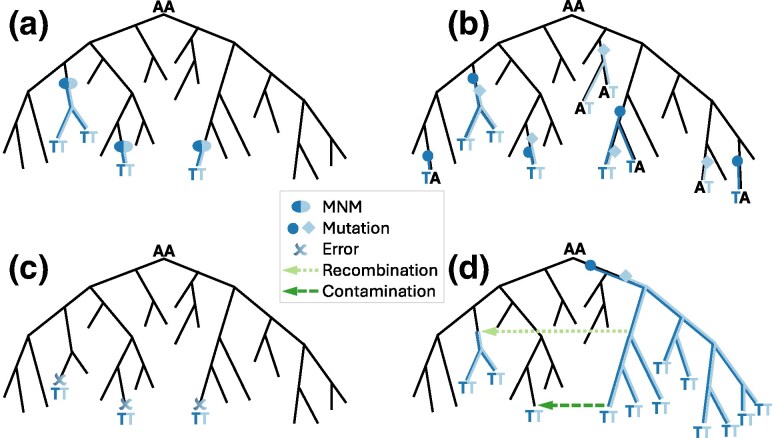
Graphical examples of causes of recurrent mnSs. We show a very small, illustrative phylogenetic tree in black. In all examples we show the substitution history of two genome positions for both of which the root nucleotide is A. Substitutions for the two positions are highlighted respectively in dark and light blue. Substitution events along the tree are highlighted with blue ellipses, circles and diamonds, their descendants are highlighted with similarly colored branches, and sequenced nucleotides different from the root “AA” are shown at the tips of the tree. a) Recurrent MNMs: example recurrent AA to TT MNM. b) Combinations of individual recurrent single-nucleotide mutations: A to T mutations at the two positions occur frequently and independently of each other; by chance, both mutations can occur on the same branch, giving rise to an AA to TT mnS that is not caused by an AA to TT MNM. c) Consensus sequence and alignment artifact: errors at both genome positions can be correlated, for example caused by the same alignment error; these result in AA to TT mnSs, at terminal tree branches only, that are not caused by AA to TT MNMs. d) Recombination, contamination and mixed infections: when an mnS has many descendants (right side of the tree), recombinations (light green dotted arrow) that transfer the mnS into a different genetic background are more likely. Contamination or mixed infections (dark green dashed arrow) can cause a similar artifactual pattern at terminal tree branches.

We consider four possible causes of highly recurrent mnSs:

- Highly recurrent MNMs (Fig. 1a). While previous studies have confirmed the occurrence of MNMs in different organisms (Hampsey et al. 1988; Nakazawa et al. 1994; Hodgkinson and Eyre-Walker 2010; Schrider et al. 2011; Chen et al. 2014; Assaf et al. 2017; Wang et al. 2020), to our knowledge, highly recurrent MNMs have not been previously reported; as such, we only consider recurrent MNMs as a plausible cause of recurrent mnSs if we can reasonably exclude all other causes below.
- Combinations of successive individual single-nucleotide mutations. Some sites of the SARS-CoV-2 genome are affected by extremely highly recurrent single-nucleotide mutations (De Maio et al. 2021, 2024). If two highly recurrent single-nucleotide mutation events independently occur on the same branch of the phylogenetic tree, they cause an mnS (Fig. 1b). If this occurs often enough, the corresponding mnS becomes highly recurrent. This scenario is discussed in Section “Combinations of highly recurrent single-nucleotide mutations”.
- Consensus sequence and alignment artifacts. Imperfections in sequencing and bioinformatics protocols can lead to recurrent errors in SARS-CoV-2 genome sequences and alignment (De Maio et al. 2020; Turakhia et al. 2020; De Maio et al. 2024). Some of these problems can simultaneously affect more than one nucleotide, and lead to recurrent mnSs in inferred mutation-annotated trees (De Maio et al. 2020; Turakhia et al. 2020; De Maio et al. 2024) (Fig. 1c). In Section “Sequence and alignment artefacts”, we discuss how we identify such artifacts by jointly analyzing sequencing read data and phylogenetic trees.
- Recombination and contamination. Recombination causes the exchange of part of the genome between different lineages (Turakhia et al. 2020; Smith et al. 2023). For recombination to occur in SARS-CoV-2, two different viral lineages need to infect the same host at the same time, and this is more likely when the two lineages are both circulating at high prevalence in the host population. If one, but not the other, lineage contains a pre-existing mnS (either due to an MNM or a combination of successive mutations), then recombination can cause a new occurrence of the same mnS (Fig. 1d). If this occurs often enough, it might cause a highly recurrent mnS. Contamination (Lagerborg et al. 2022) (multiple lineages accidentally mixed in the same sample) and mixed infections (Rockett et al. 2022) can cause a similar observed pattern: if the two lineages present in the sample have varying sequencing read depth along the genome, and especially if an amplicon dropout affects only some of the two lineages, it can result in a mixed consensus sequence, that effectively looks like a recombinant (Fig. 1d). Recombination and contamination are discussed in Section “Recombination and contamination”.

In Table 1, recurrent mnSs that we find unlikely to be caused by genuine recurrent MNMs are highlighted in bold. Each is discussed below.

### Combinations of Highly Recurrent Single-nucleotide Mutations

Substitution rate variation along the genome can increase the number of expected mnSs (Smith et al. 2003). In SARS-CoV-2, extremely highly recurrent single-nucleotide mutations (De Maio et al. 2021, 2024) are indeed expected to cause recurrent mnSs when pairs of these mutations occur by chance on the same phylogenetic tree branch (Fig. 1b).

In our dataset, the most highly recurrent single-nucleotide substitutions are G11083T (11,206 substitutions) and C21575T (6,913 substitutions); their combination, G11083T-C21575T, with 53 occurrences, is the most recurrent mnS composed of non-neighboring substitutions (Table 1, mnS4). It is reasonable to conclude that occurrences of mnS4 are likely combinations of independently occurred single-nucleotide mutation events. Permutation tests (see Methods) suggest that indeed such a number of occurrences of mnS4 is to be expected just by chance, while the same is not true for any other mnS more recurrent than mnS4 (see column “Occurrences in simulations” in Table 1). For this reason, from here onward we exclude mnS4 from consideration as a possible recurrent MNM and focus only on more-frequent mnSs.

**Fig. 2.**
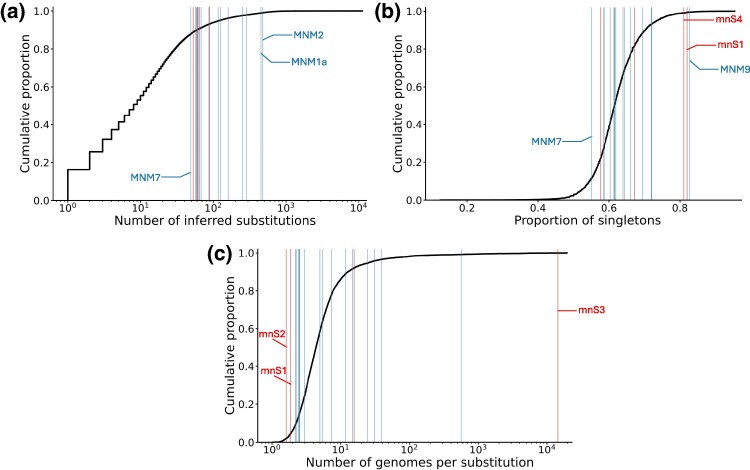
Comparisons between recurrent mnSs and single-nucleotide substitutions in SARS-CoV-2. We show in black cumulative distributions for single-nucleotide substitutions. For any genome position, only the three substitutions away from the reference nucleotide are considered, and of these only those that are inferred to have occurred at least once (57,228 substitutions out of all possible 89,709). Red vertical bars correspond to mnSs that are unlikely to be caused by MNMs (red entries in Table 1), blue bars to all other mnSs in that table. a) Numbers of substitutions, as in Table 1. mnSs in Table 1 are more recurrent than most single-nucleotide substitutions, since the left-most blue bar (corresponding to MNM7, which has 49 occurrences, see *x*-axis) intersects the black cumulative distribution at a value of around 0.88, meaning that the least recurrent mnS in Table 1 is more recurrent than 88% of single-nucleotide substitutions observed in our dataset. b) Proportions of substitutions that are singletons (i.e. occurring on terminal branches of the phylogenetic tree and therefore inherited by only one sampled genome), like column “Proportion of singletons” in Table 1. Substitutions due to recurrent consensus sequence errors are typically enriched in singletons (they are represented at the right-hand side of the plot), since each individual error event is not inherited and therefore only affects one sample. This is for example the case for the right-most red vertical bar in the plot, corresponding to mnS1 which we argue is caused by sequencing data processing issues (see Section “Proportion of singletons”). While MNM9 is similarly enriched in singletons, we do not find any other evidence suggesting that MNM9 is problematic, and we therefore think that the enrichment in singletons for MNM9 might be due to this mutation being slightly deleterious (see Section “Proportion of singletons”). c) Number of observed genomes containing the considered substitution, divided by the number of occurrences of that substitution along the phylogenetic tree; that is, the average number of sampled descendants of a substitution event (like column “Observed genomes per occurrence” in Table 1). Substitutions due to recurrent consensus sequence errors are expected to have few genomes per substitution (as we suggest is the case for mnS1 and mnS2). Recurrent substitutions attributable to recombination or reference biases are expected to have many descendant genomes per substitution, as we suggest is the case here for mnS3 (see Section “Read data for cherries”).

All these remaining recurrent mnSs are composed of much rarer single-nucleotide substitutions than G11083T or C21575T (column “Individual occurrences” in Table 1). In fact, these mnSs are often more recurrent than the individual single-nucleotide substitutions they are composed of, and often involve more than two nucleotide substitutions. These mnSs are also more recurrent than the vast majority of single-nucleotide substitutions in the SARS-CoV-2 genome (Fig. 2a), with MNM1a and MNM2 being among the most highly recurrent (either single-nucleotide or multinucleotide) substitutions overall. It is therefore extremely unlikely that any substantial proportion of the occurrences of these remaining mnSs is the result of independently occurring single-nucleotide mutation events, as confirmed both by permutation and hypergeometric tests (see Methods and Table 1). In particular, across all permutations performed, no mnS comprising neighboring substitutions (⩽10 bp apart) ever occurred more than 50 times; the most recurrent was C11074T-G11083T, which occurred a maximum of 22 times in any individual permutation replicate, and which is composed of highly recurrent mutations (Supplementary Table S4).

As noted above, we did not investigate mnSs less recurrent than mnS4, since these are expected to have a less impact on phylogenetic analyses, are harder to confirm as MNMs, and seem often to be the result of coincidences of separate mutations. We report however the remaining mnSs with at least 20 occurrences, as follows. Those that cannot be explained as combinations of highly recurrent single-nucleotide mutations are included in Supplementary Table S3. We did not investigate which of these might be attributable to sequence artifacts and which to recurrent MNMs. Recurrent mnSs that are combinations of highly recurrent single-nucleotide mutations are instead listed in Supplementary Table S4. One exception is mnS C11950T-C28472T which has 21 occurrences and is composed of relatively rare single-nucleotide substitutions (172 total occurrences of C11950T and 232 of C28472T). This makes it highly unlikely to be the result of a combination of independently occurring single-nucleotide substitutions (hypergeometric test *P*-value $10^{-63}$, Bonferroni-corrected $8\cdot 10^{-54}$, 0–1 occurrences expected from the permutation test). However, C11950T-C28472T is composed of nucleotide substitutions that are distant in the genome, and as such we currently have no explanation for its recurrence.

### Sequence and Alignment Artefacts

Artefacts in consensus genome calling and in alignments can cause mnSs (De Maio et al. 2020; Turakhia et al. 2020; De Maio et al. 2024). Following De Maio et al. (2024), we use a combination of three approaches to identify instances of these issues in our SARS-CoV-2 data set:

- We investigate the proportions of singletons (substitutions occurring on terminal branches of the phylogenetic tree) for different types of substitution (Section “Proportion of singletons”). While a true mutation can be inherited by many descendants, each consensus calling error only affects one sample (see Fig. 1c), usually resulting in an enrichment of singleton substitutions at positions with recurrent consensus sequence errors (De Maio et al. 2024). An enrichment in singletons is however not sufficient on its own to assess that a recurrent mnS is due to a recurrent error, since deleterious mutations are also expected to show an enrichment in singletons.
- We analyze sequencing read data (Section “Read data for cherries”). Recurrent consensus sequence errors are often associated with low sequencing depth, high heterozygosity in the raw sequencing reads, and indels (De Maio et al. 2024). For each recurrent mnS considered, we systematically analyze sequencing read data of “cherries” (sibling pairs of samples in the phylogenetic tree) in which only one of the two siblings contains the mnS. In these situations, the sibling not containing the mnS shows the genomic background in which the mnS has occurred. For each such cherry, we investigate sequencing reads that map near the considered mnS (see Methods). Sequencing read data from cherries can highlight for example if an mnS can be explained by read alignment errors near a pre-existing indel, by reference biases, or by incomplete primer trimming.
- We investigate phylogenetic clustering (Section “Phylogenetic clustering”). A given recurrent error can preferentially appear in certain parts of the phylogenetic tree if, for example, it is caused by a particular sequencing approach or genomic background (De Maio et al. 2020; Turakhia et al. 2020; De Maio et al. 2024). We therefore consider phylogenetic clustering as a warning flag for consensus sequence errors; we do not consider it as a sufficient condition to rule an mnS as artifactual, however, since phylogenetic clustering can also be caused by context-dependent mutational events.

#### Proportion of Singletons

Only three of the recurrent mnSs considered present unusually high proportions of singletons (Fig. 2b and Table 1): mnS4 (which we already discarded above), mnS1, and MNM9. mnS1 is indeed typically found in regions of sudden drop in sequencing depth (Supplementary Table S1) and appears to be the result of consensus sequence calling errors due to incomplete read trimming. As such, we also consider mnS1 to be problematic and unlikely the result of recurrent MNMs.

MNM9 is also substantially enriched in singletons, but we found no other evidence to suggest that it is caused by artifacts (see sections “Read data for cherries” and “Phylogenetic clustering”). For this reason, it is possible that MNM9 (which causes an amino acid substitution in gene M, see Table 1 and Fig. 7) is caused by a genuine but deleterious recurrent MNM, reducing the ability of the virus to spread and infect new hosts.

#### Read Data for Cherries

Recurrent heterozygosity in read data is often the hallmark of recurrent sequencing errors (De Maio et al. 2020; Turakhia et al. 2020; De Maio et al. 2024) but apart from mnS1 and mnS4, which we already identified as problematic, no other mnS is highly enriched in heterozygosity in sequencing read data (column “Heterozygosity in cherries” in Table S2).

Recurrent consensus sequence errors can also emerge in regions of low read depth (e.g. due to systematic amplicon dropout). mnS2 is the only recurrent mnS appearing to be enriched in regions of reduced sequencing depth (<100 in 26/28 cherries considered, see column “Low coverage in cherries” in Table S2). Indeed, mnS2 appears to be an artifact almost entirely specific to Alpha lineage genomes (Supplementary Fig. S1A) caused by a pre-existing common deletion at neighboring positions 21991-21993, local sequence similarity (Supplementary Fig. S1B), and drops in sequencing depth (Supplementary Fig. S1C), causing read alignment and consensus calling errors. mnS3 (comprising mutations T28881A-G28882A-G28883C) often appears within a genomic background containing a deletion at these positions (12 out of 16 cherries). While we avoid most reference biases in consensus sequence calls by using Viridian (Hunt et al. 2024; in particular, we prevent calling reference nucleotides at positions with very low coverage), we may re-introduce some of this bias by masking short deletions (in this case, replacing gap characters with reference genome nucleotides) to improve phylogenetic inference (De Maio et al. 2024). Most inferred substitution events of T28881A-G28882A-G28883C, which is the most prevalent mnS among sampled genomes (column “Number of observed genomes” in Table 1), appear to be caused by this reference bias. mnS3 seems to be the only recurrent mnS affected by this problem since, due to its abundance, the nucleotides AAC at positions 28881–28883 are the genome-alignment-wide consensus nucleotides used for masking short deletions. In the following we consider this mnS as problematic and unlikely to be caused by recurrent MNMs.

None of the remaining mnSs systematically occurs near a pre-existing background indel. Instead, MNM4, MNM7, MNM7a, and MNM10 appear to consistently introduce certain indels (column “Deletions in cherries” in Table S2), specifically 1–3 nucleotide deletions, not present in the genomic background on which they occur (Supplementary Fig. S2). Since we find no other evidence to suggest these mnSs are artifactual, we suggest that these mnSs and deletions are the result of complex MNM events that not only substitute 2–4 nucleotides, but also consistently shorten the genome by 1–3 nucleotides.

Visual examination of read assembly data does not reveal any clear sequencing data issues at mnSs not identified as problematic (Supplementary File S1).

#### Phylogenetic Clustering

Phylogenetic clustering of substitution events can be a warning flag for recurrent errors (for example highlighting errors caused by certain sequencing protocols De Maio et al. 2024), but it can also emerge naturally as mutation rates might strongly depend on the background nucleotide context (De Maio et al. 2021, 2024). As discussed in Section “Read data for cherries”, phylogenetic clustering is observed for the problematic mnS2 (Supplementary Fig. S1A), linked to the presence of a background deletion causing read alignment errors.

**Fig. 3.**
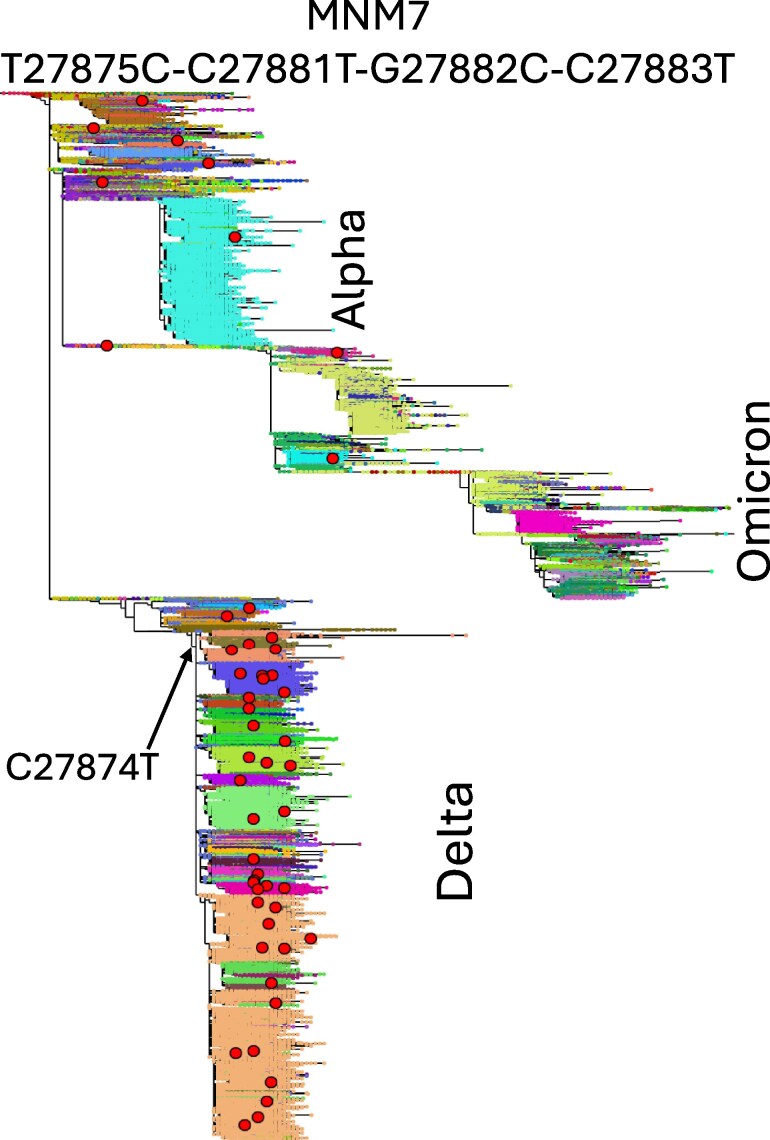
Phylogenetic distribution of MNM7. We show our background SARS-CoV-2 phylogenetic tree relating >2 million genomes (De Maio et al. 2024) where sampled genomes, represented by small dots, are colored according to their Pango (Rambaut et al. 2020) lineage (assigned by Pangolin V4.3 with pangolin-data V1.21; O’Toole et al. 2021). Occurrences of MNM7 are highlighted with larger red circles. These appear enriched in the Delta lineage, where neighboring substitution C27874T (arrowed) is ancestral to most genomes. The tree and substitutions are visualized with Taxonium (Sanderson 2022).

**Fig. 4.**
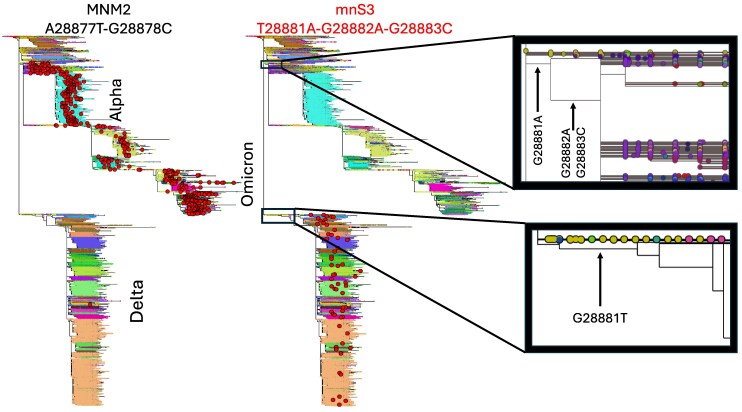
Phylogenetic distribution of MNM2 and mnS3. Details are as in Fig. 3. Left: occurrences of MNM2 are abundant in Alpha and Omicron lineages, but not in Delta. Right: occurrences of mnS3. While most occurrences of mnS3 in Delta are likely to be artifacts (see Section “Read data for cherries”), we do not challenge the genuineness of the G28881A, G28882A and G28883C substitutions ancestral to the Alpha and Omicron lineages (top zoom-in box on the right), which cause most Alpha and Omicron genomes (in which most MNM2 occurrences are observed) to have nucleotides AAC at positions 28881–28883.

We only observe obvious phylogenetic clustering for two other mnSs, MNM7 (Fig. 3) and MNM2 (Fig. 4; the phylogenetic distribution of the other mnSs is shown in Supplementary Fig. S3). Occurrences of MNM7 (but not MNM7a) seem to particularly appear in the background of the neighboring substitution C27874T (Fig. 3). Similarly, MNM2 appears to preferentially occur in the mutated background containing the nearby mnS3 (Fig. 4). Unlike for mnS2, we find no other evidence suggesting that MNM7 and MNM2 are not caused by genuine MNMs. This suggests that MNMs causing these mnSs might be preferentially happening in a specific nucleotide context; this is further explored in Section “Mechanisms of recurrent MNMs”.

### Recombination and Contamination

Homologous recombination can cause the recurrence of an mnS by transferring genetic material from a lineage with the mnS to a lineage without it (Fig. 1d). Such recombination-driven recurrences are expected to be more common for high-frequency mnSs (those that are present in many genomes), since recombination requires a host to be simultaneously infected by two viral lineages, one containing the mnS and one not containing it. Sample contamination and mixed infections can lead to a similar pattern (but only at the terminal branches of the phylogenetic tree, see Fig. 1d) in cases when the consensus genome is a hybrid between genomes in the sample. This can occur if the two genomes occur in similar proportion, or if they are differently affected by variation in sequencing depth (for example if at a given position the main genome is affected by amplicon dropout but the minor genome is not). These effects of contamination and mixed infections are also expected to be more common for high-prevalence variants, since a high-prevalence variant is more likely to be included in a sample as contaminant or as part of a mixed infection.

Unless these mnSs themselves would cause extremely high rates of recombination/contamination (which seems unlikely), then the rate of substitutions caused by recombination/contamination should depend only of the prevalence of the substitution, and substitutions with similar prevalence should have similar numbers of substitutions caused by recombination/contamination. Because recurrent mnSs have similar prevalence to single-nucleotide substitutions in SARS-CoV-2 (Fig. 2c), we expect a similar number of recombination-induced substitutions for them as for single-nucleotide substitutions. However, recurrent mnSs show higher numbers of substitutions compared to single-nucleotide substitutions (Fig. 2c), and previous analyses suggest that recombination is not the leading cause of substitutions in SARS-CoV-2 (see e.g. Turakhia et al. 2022). It is therefore not plausible that recombination or contamination are the main driver of recurrent mnSs.

The only two recurrent mnSs with high prevalence are mnS3 and MNM3. Of these, mnS3 is in particular an ideal candidate for homologous recombination and contamination, given that it is present in close to half of the SARS-CoV-2 genomes. However, we only inferred 59 occurrences of mnS3, the majority of which appear to be artifacts caused by deletion masking (12 out of 16 cherries, see Section “Read data for cherries”), not recombination or contamination. This further corroborates that it is unlikely that homologous recombination or contamination cause a substantial proportion of observed recurrent mnSs.

Assuming that homologous recombination and contamination events would not favor exchange of one genotype over the other, that is, they are symmetrical with respect to the parent genomes (note however that several factors might invalidate this assumption at certain positions, like context-dependent recombination rates or amplicon drop-out), one would expect them to cause a similar number of occurrences of a certain mnS and its reverse. For example, considering A28881T-A28882G-C28883G as the reverse of mnS3 (and also considering A28881G-A28882G-C28883G as its reverse, due to the high prevalence of nucleotide G at position 28881: Fig. 4), in total we inferred just 9 reversions of mnS3. In general, very few reversions were inferred for all recurrent mnSs (Table S2 column “Reversions”), further reducing the likelihood of a substantial impact from homologous recombination and contamination. For the other high-prevalence mnS, MNM3, we inferred only 5 reversions, in contrast with its 253 inferred occurrences. All these observations suggest that recombination and contamination are not the main cause of the recurrent mnSs considered.

### Selection

Compensatory mutations or positive selection can also lead to mnSs (Bazykin et al. 2004; Belinky et al. 2019). Given that SARS-CoV-2 phylogenetic tree branches are very short, that many highly recurrent mnSs are composed of >2 nucleotide substitutions, and that many individual single-nucleotide mutations composing these mnSs are not highly recurrent, it is unlikely that positive selection affecting individual single-nucleotide mutations or compensatory mutations contribute substantially to the recurrence of these mnSs. It is also unlikely that the identified recurrent MNMs are themselves under strong positive selection (and therefore that positive selection might be causing their recurrence), given that they give rise to similar proportions of singletons (Fig. 2b) and descendants per mutation (Fig. 2c) as typical single-nucleotide mutations in the SARS-CoV-2 genome, and so appear to be under similar selective pressure.

The recurrent mnSs most compatible with a causative mechanism attributable to selection acting on separate successive 1-nt mutations (e.g. strong compensatory or epistatic selection) are those involving just two nucleotide substitutions and no indels, and composed of exactly two nonsynonymous substitutions (we assume it unlikely a synonymous substitution would be under strong selection). Out of the 7 candidate recurrent MNMs identified involving just two nucleotide substitutions, four are composed of two nonsynonymous substitutions (MNM1a, MNM2, MNM5, and MNM13), while the remaining three involve at least one synonymous substitution (MNM6, MNM11 and MNM12). This proportion (4/7, 57.1%) is similar and not significantly higher than the one expected from random pairs of mutations (54.2%; *P*-value 0.59, see Methods). Similarly, these 7 recurrent MNMs are composed of a total of 10 nonsynonymous substitutions and 4 synonymous ones and this 10/14 proportion (71.4%) is lower than the 76.0% proportion expected from random pairs of mutations (and in particular is not significantly higher, *P*-value 0.77, see Methods). These observations therefore provide no evidence to support possible selective mechanisms causing a substantial proportion of the observed recurrent mnSs.

### Mechanisms of Recurrent MNMs

**Fig. 5.**
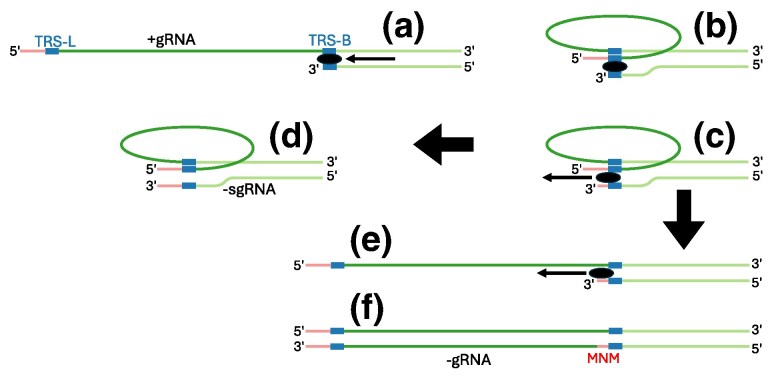
Interrupted template switching mechanism for recurrent MNMs. We sketch a possible mechanism to explain the origin of most recurrent MNMs found in this study. a) RNA-dependent RNA polymerase (RdRp, black oval) starts synthesizing a negative sense copy of the viral genome, and pauses at a TRS-B. b) Template switching is initiated, RdRp is moved to the TRS-L, and c) starts transcribing nucleotides $5^{\prime }$ of the TRS-L. After this, two scenarios can occur: either d) template switching and transcription are completed, resulting in a –sgRNA, or template switching is interrupted, and e) RdRp returns to the original TRS-B and continues transcription from there. f) If, after this, no further template switching occurs, RdRp completes replication of a −gRNA containing an MNM just $5^{\prime }$ of a TRS-B.

Template switching is used by coronaviruses and most *Nidovirales* viruses during transcription to create a set of nested negative strand subgenomic RNAs (−sgRNAs), see V’kovski et al. (2021), Malone et al. (2022) and Bradley et al. (2024). During template switching, negative strand synthesis stops at one of several transcription regulatory sequences (TRS-B, see Fig. 5a) and is re-initiated at the leader TRS (TRS-L) near the $5^{\prime }$ end of the genome (Fig. 5b, c). After the transcription of the −sgRNA is completed (Fig. 5d), this is then used to synthesize a corresponding +sgRNA, which in turn is translated into a structural or accessory protein (V’kovski et al. 2021; Malone et al. 2022; Bradley et al. 2024). In SARS-CoV-2, the consensus TRS motif is $5^{\prime }$-ACGAAC-$3^{\prime }$ and template switching may be initiated at any of several TRS-Bs along the genome. This results in −sgRNAs of different lengths, and this process is thought to modulate gene expression (V’kovski et al. 2021; Malone et al. 2022; Bradley et al. 2024). If template switching does not happen at any TRS-B, transcription results in replication of a full genomic negative sense RNA (–gRNA), which is used as a template to synthesize +gRNA, which in turn can be used for translation or be packaged into new virions.

Template switching (either TRS-induced or TRS-independent) is also considered one of the main causes of indels, recombination, and noncanonical transcripts (uncommon –sgRNAs resulting from template switching from nonstandard TRSs) in SARS-CoV-2 (Kim et al. 2020; Chrisman et al. 2021; Garushyants et al. 2021; Bradley et al. 2024; Liang et al. 2022; Ugolini et al. 2022), and it therefore represents a reasonable potential mechanism causing recurrent MNMs.

We find strong links between the majority of the highly recurrent MNMs, and the $5^{\prime }$-ACGAAC-$3^{\prime }$ TRS-L motif, suggesting a connection between many recurrent MNMs and template switching. More specifically, we find that recurrent MNMs seem to often occur within just a few base pairs $5^{\prime }$ of a consensus TRS motif (6 out of 14 MNMs), or convert a sequence similar to a consensus TRS motif into a consensus one (more details are given below). We also find that the TRS-L region is a suitable template for almost every recurrent MNM (13 out of 14). Following this evidence, we suggest that most highly recurrent MNMs in SARS-CoV-2 might be caused by interrupted template switching during viral replication (see Fig. 5c, e). We suggest that in this process template switching induces a few bases from the TRS-L and/or $5^{\prime }$ of the TRS-L to be transcribed, after which the template switching is interrupted and transcription is restarted from near the original TRS-B. If template switching does not happen for the rest of the transcription process, this results in a –gRNA with an MNM just $5^{\prime }$ of the TRS-B involved (Fig. 5f).

**Fig. 6.**
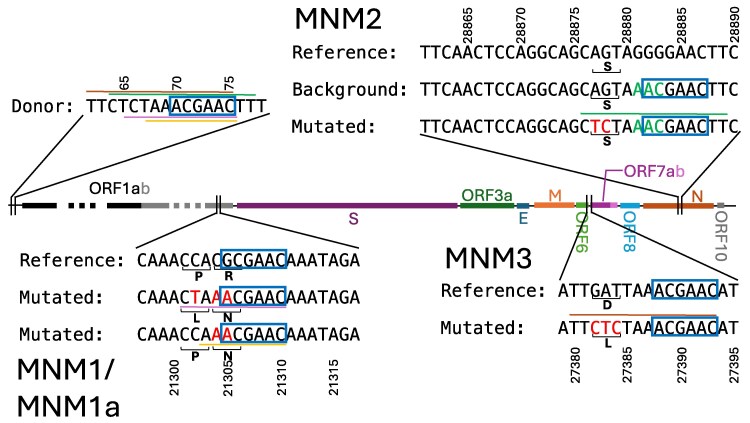
Suggested origin of the four most recurrent MNMs: MNM1, MNM1a, MNM2 and MNM3. TRS motifs are highlighted with blue boxes. Center: Representation of the SARS-CoV-2 genome, annotated with its genes. Top left: $5^{\prime }$ UTR reference genome sequence including the TRS-L (“Donor”). Top right: Representation of MNM2 (A28877T-G28878C), showing the reference sequence near the MNM, the background sequence that is typically mutated by the MNM (the differences between the reference and the background are highlighted in green), and the sequence resulting from the MNM (“Mutated”) with substituted nucleotides highlighted in red. Note that the background sequence contains the TRS motif, while the reference sequence does not (Fig. 4), which we suggest is the reason why the MNM mostly occurs in this background. Sequence identity between the mutated sequence and the TRS-L region is highlighted with a green line. Bottom right: Representation of MNM3 (G27382C-A27383T-T27384C). Sequence identity with the TRS-L region is highlighted with a brown line. Bottom left: Representation of MNM1 and MNM1a (C21302T-C21304A-G21305A and C21304A-G21305A). Amino acids affected by the MNMs are shown under the nucleotide sequences. Sequence identity with the TRS-L region is highlighted with purple and yellow lines.

**Fig. 7.**
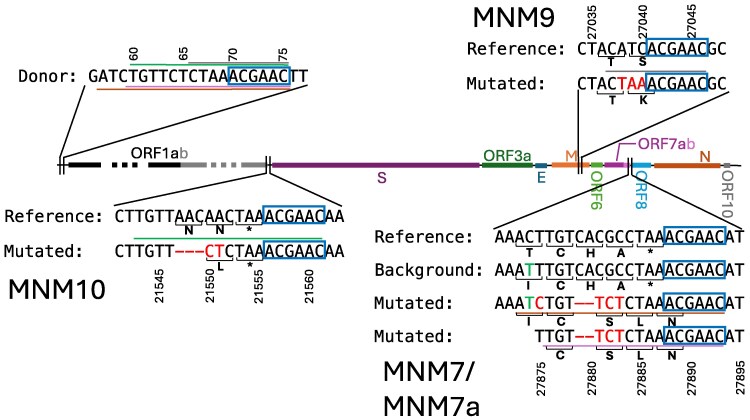
Suggested origin of MNM9, MNM10, MNM7 and MNM7a. Details are as in Fig. 6. The 2-nt deletions observed jointly with MNM7 and MNM7a cause a frameshift in ORF7b that likely renders it nonfunctional.

Six MNMs (MNM2, MNM3, MNM7, MNM7a, MNM9 and MNM10) fit this suggested mechanism well (Figs. 6 and 7), occurring 1–5 bp $5^{\prime }$ of a consensus TRS motif (binomial test *P*-value $10^{-13}$: see Methods section). In particular, the highly recurrent MNM2 almost exclusively happens in a genetic background containing the consensus TRS motif ACGAAC nearby (Fig. 6, cf. Fig. 4), providing further evidence of the importance of TRSs to highly recurrent MNMs.

Another seven MNMs (MNM1/1a, MNM5, MNM6, MNM8, MNM11 and MNM12) are consistent with the proposed mutational mechanism. They occur just $5^{\prime }$ of a sequence similar, though not identical, to the consensus TRS motif (GCGAAC, TTGAAC, AAGAAC, GCAAAC, GCGAAC, and ACGATC, respectively; see Supplementary Figs. S4 and S5). Also, some of these seven MNMs usually (MNM1/1a) or sometimes (MNM5, MNM11) or partially (MNM8) create a consensus TRS motif, again consistent with our proposed mutational mechanism, and suggesting that interrupted template switching might help maintain existing and create new consensus TRS motifs in the genome. We note also that MNM1a and MNM7a differ from their larger versions MNM1 and MNM7 at the $5^{\prime }$ ends of their respective mutation clusters (and thus at the $3^{\prime }$ ends of the −gRNA: see Fig. 5), consistent with these nested MNMs being formed by earlier return to the original TRS-B under our proposed mechanism. Finally, all substitutions and indels involved in these 13 MNMs consistent with interrupted template switching create or increase sequence identity with the TRS-L and the region just $5^{\prime }$ of it (Figs. 6, 7, S4 and S5), as expected under the proposed mechanism.

MNM4 occurs at the $3^{\prime }$ side of a consensus TRS motif (Supplementary Fig. S4) instead of the usual $5^{\prime }$ side, and as such we do not consider it as a good fit for our proposed mutational mechanism. In total, 14 of the 15 MNMs we have identified are associated with TRS motifs. Of these, six fit our proposed mutational mechanism perfectly while seven fit it imperfectly as they involve a sequence almost, but not completely identical to the consensus TRS motif; between them these 13 MNMs account for 91% (2,187/2,404) of the individual occurrences of MNMs listed in Table 1.

We cannot exclude that a different mechanism other than interrupted template switching might be the cause of at least some of these MNMs, and we do find, for some of them, other regions of sequence identity within the SARS-CoV-2 genome (Supplementary Fig. S6). This is particularly relevant for MNM13, which is the only recurrent MNM considered here for which we found no obvious links to TRSs and template switching (Supplementary Fig. S6G). A possible alternative mechanism causing recurrent MNMs is sequence-directed mutagenesis (Dutra and Lovett 2006; Ladoukakis and Eyre-Walker 2007) which seemingly involves accidental template switching between nearby imperfect inverted repeats (rather than programmed long-range template switching as in our proposed mutational mechanism). However, we did not find nearby imperfect inverted repeats (see Methods section) that could explain any of the considered highly recurrent MNMs.

### Impact of Recurrent MNMs on the Inference of Recombination

Just as homologous recombination can cause mnS occurrences (Fig. 1d), so recurrent MNMs might be misinterpreted as the result of homologous recombination events, since recurrent MNMs cause the same cluster of nearby substitutions to appear in different lineages. We typically model nucleotide substitutions as single-nucleotide independent events, and clustered substitutions within a small genomic region and within a phylogenetic branch are considered the hallmark of detectable homologous recombination events (Didelot and Wilson 2015; Turakhia et al. 2022). This same pattern is however also caused by recurrent MNMs. A single recurrent MNM therefore might, on its own, generate sufficient signal to cause the spurious inference of many recombination events deemed statistically significant.

To test this, we investigated recombination events inferred by RIVET (Turakhia et al. 2022; Smith et al. 2023) from global SARS-CoV-2 genomes collected up to June 2024 (Smith et al. 2023). Out of a total of 13,204 inferred recombination events, 1,579 (12%) were exclusively informed by one of the recurrent MNMs from Table 1. We therefore suggest that these are false positives, and that about one out of every 7 inferred recombination events in SARS-CoV-2 evolution is likely the result of a recurrent MNM. In general, one would expect larger MNMs (those causing more simultaneous nucleotide substitutions) to generate more significant recombination event false positives. Indeed, since RIVET uses a parsimony score threshold of 3 to infer recombination events, 2-nt MNMs on their own do not lead to recombination event inference in RIVET, and did not contribute to any of the 1,858 recombination events informed exclusively by recurrent MNMs (Table 2).

**Table 2.** Number of recombination events inferred by RIVET involving recurrent mnSs.

ID	mnS	Recombinations	Recombinations entirely
		involving mnS	defined by mnS
MNM1	C21302T	332	325
	-C21304A		
	-G21305A		
> MNM1a	C21304A	6	
	-G21305A		
MNM2	A28877T	112	
	-G28878C		
MNM3	G27382C	850	575
	-A27383T		
	-T27384C		
MNM4	T26491C	261	206
	-A26492T		
	-T26497C		
MNM5	G27758A	0	
	-T27760A		
MNM6	C25162A	3	
	-C25163A		
MNM7	T27875C	52	50
	-C27881T		
	-G27882C		
	-C27883T		
> MNM7a	C27881T	41	16
	-G27882C		
	-C27883T		
MNM8	T21294A	320	287
	-G21295A		
	-G21296A		
**mnS1**	**A507T**	0	
	**-T508C**		
	**-G509A**		
MNM9	A27038T	130	120
	-T27039A		
	-C27040A		
MNM10	A21550C	0	
	-A21551T		
**mnS2**	**T21994C**	9	
	**-T21995C**		
MNM11	C13423A	24	
	-C13424A		
MNM12	A4576T	11	
	-T4579A		
**mnS3**	**T28881A**	542	279
	**-G28882A**		
	**-G28883C**		
MNM13	A20284T	2	
	-T20285C		
**mnS4**	**G11083T**	0	
	**-C21575T**		

As in Table 1, we mark in bold mnSs that are unlikely to be caused by recurrent MNMs.

Recombinations involving mnS: numbers of recombination events containing each mnS within the recombinant genetic material. These are all the inferred recombinations that might have been influenced by recurrent mnSs.

Recombinations entirely defined by mnS: number of inferred recombination events exclusively informed by the considered mnS (no other SNP is inferred to have been exchanged during recombination). We consider these events as recombination false positives caused by MNMs (except for the row corresponding to mnS3). Recombinations in this column are also included in the second column. Two-nt mnSs are not annotated in that column since RIVET uses a threshold of three substitutions for inferring recombination.

Recurrent multinucleotide consensus sequence errors, just like recurrent MNMs, might also lead to recombination inference false positives. Unlike the SARS-CoV-2 datasets we used, the genome sequences used by RIVET are not called with Viridian. For this reason, issues with reference biases mentioned earlier regarding mnS3 might be more pronounced in the RIVET dataset, and are probably the cause of the large number of inferred recombination events for this mnS in Table 2 (279, 2.1% of all inferred recombinations).

These analyses show that recurrent MNMs and recurrent multinucleotide errors, if unaccounted for, greatly and adversely affect the inference of homologous recombination and cause high rates of false positives in SARS-CoV-2. However, recurrent MNMs and multinucleotide errors can also be flagged either by using Table 1, or, more generally, by classifying as unreliable all recombinations informed by clusters of substitutions <10 bp apart. We have now implemented both flags within RIVET v0.2.0 (https://github.com/TurakhiaLab/rivet).

## Discussion

The existence and prevalence of MNMs has long been debated in the scientific literature (Golding and Glickman 1985; Hampsey et al. 1988; Nakazawa et al. 1994; Averof et al. 2000; Smith et al. 2003; Whelan and Goldman 2004; Hodgkinson and Eyre-Walker 2010; Schrider et al. 2011; Chen et al. 2014; Assaf et al. 2017; Wang et al. 2020). MNMs allow evolution to explore areas of genome space that are less reachable through single-nucleotide substitutions alone (Averof et al. 2000), and substantially contribute to human disease (Kaplanis et al. 2019; Wang et al. 2022). MNMs are also important in computational biology where they can detrimentally impact data analyses including annotation and diagnosis (Kaplanis et al. 2019; Wakeling et al. 2019; Degalez et al. 2021; Srinivasan et al. 2021; Wang et al. 2022) and selection scans (Venkat et al. 2018; Dunn et al. 2019; Lucaci et al. 2021, 2023) since most methods wrongly interpret MNMs as combinations of separate single-nucleotide substitutions.

Previous studies have assumed that MNMs are rare and homogeneously distributed along the genome (De Maio et al. 2013; Dunn et al. 2019; Lucaci et al. 2021, 2023). However, our analysis of millions of SARS-CoV-2 genomes shows the existence of several highly recurrent MNMs. The impact of highly recurrent MNMs in computational biology is considerably more significant than that of homogeneously distributed MNMs. For example, highly recurrent MNMs are expected to greatly compound the problem of false positives in positive selection scans, causing false positives not only with branch-site tests (Yang and Nielsen 2002), but also with the generally more robust site-based tests (Nielsen and Yang 1998). The most striking example of this is probably the synonymous serine-to-serine MNM2 (A28877T-G28878C) (Fig. 6), which is composed of two nonsynonymous single-nucleotide substitutions at the same codon. This MNM is inferred to have occurred 480 times (Table 1), but there are only <250 other substitutions in total at this codon. So, while the majority of mutations at the codon are synonymous when modeling MNMs, if we model only single-nucleotide mutations we would infer that the vast majority of the substitutions at the codon are nonsynonymous, which is likely to greatly affect the inference of selective pressure at this codon (Lucaci et al. 2023).

As shown in Section “Impact of recurrent MNMs on the inference of recombination”, highly recurrent MNMs also adversely affect the inference of recombination. Although our results are based on inference by RIVET (Turakhia et al. 2022; Smith et al. 2023), we expect virtually all existing recombination detection methods to be affected by this issue.

Phylogenetic tree inference can also be negatively impacted by recurrent MNMs, since sequences descending from different occurrences of the same MNM could be wrongly clustered together and be interpreted as monophyletic due to the shared MNM. This is similar to the phylogenetic issues caused by convergent evolution (Zou and Zhang 2016), and might indeed be affecting our own analyses, leading to underestimation of the number of occurrences of recurrent MNMs.

We propose a mechanism of interrupted template switching as a plausible leading cause of recurrent MNMs in SARS-CoV-2. This mechanism is consistent with the known process of transcription and replication of SARS-CoV-2 (V’kovski et al. 2021; Malone et al. 2022; Bradley et al. 2024). In particular we demonstrate the association of highly recurrent MNMs with consensus TRS motifs, or with similar motifs. In these latter cases, we note that the MNM often partially or wholly converts the motif to a consensus TRS (MNM1, MNM1a, MNM5, MNM8, MNM11, see Fig. 6 and Supplementary Figs. S4 and S5). This is consistent with the RdRp backtracking process implicated in SARS-CoV-2 template switching (Chen et al. 2020) and the possibility of subsequent error correction using the TRS-L motif as template (Robson et al. 2020), providing further circumstantial evidence for our proposed mechanism. Since all coronaviruses (as well as other nidoviruses) share a similar template switching mechanism with SARS-CoV-2 (Pasternak et al. 2006), it is fair to assume that their evolution is probably also affected by recurrent MNMs. We think it is an important open question to find which other organisms outside the *Nidovirales* also experience recurrent MNMs. As more genomic data is produced and shared for a large number of pathogens, we will not only be more able to address this question, but also be able to improve genomic analyses by accounting for identified recurrent MNMs.

As discussed in Section “Selection”, it is unlikely that positive or compensatory selection contribute substantially to our findings. However, mutations caused by our proposed mechanism of interrupted template switching naturally increase the similarity between the affected genomic region and the TRS-L sequence, and therefore tend to create new TRSs or make existing TRSs more similar to the consensus TRS (see Mears et al. 2025 for examples of recurrent MNMs creating new TRS-B sequences and affecting the sgRNA spectrum of SARS-CoV-2). While TRSs are often under strong purifying selection (Haddox et al. 2025), our results show that we cannot assume that the convergent creation of new TRSs is necessarily the result of positive selection favoring them (Mears et al. 2025), since the neutral mutational bias caused by interrupted template switching can have the same effect.

The public availability of millions of SARS-CoV-2 genomes has made it possible to track the evolution of the virus in great detail during the COVID-19 pandemic. Without so many genomes, we would not have been able to identify highly recurrent MNMs, and without publicly available sequencing read data (Harrison et al. 2021) we would not have been able to validate them. In the future, we expect that further increases in sequencing capacity and genome data sharing will allow us to similarly track and understand the evolution of many other pathogens.

## Methods

We investigated a SARS-CoV-2 dataset containing 2,072,111 genomes collected up to February 2023. Consensus sequences were called with Viridian (Hunt et al. 2024), a tool that prevents common reference biases in genomic regions of low sequencing coverage. To reduce the number of contaminated samples, we removed from the dataset genomes enriched in within-sample genetic diversity, as described in De Maio et al. (2024). We then masked alignment columns affected by previously identified recurrent consensus sequence errors, and estimated a phylogenetic tree using MAPLE v0.6.8 (De Maio et al. 2024) with an UNREST substitution model, rate variation, and deep SPR phylogenetic search. For a full description of data preparation and phylogenetic inference, see De Maio et al. (2024). We then kept track of all substitutions inferred along the phylogenetic tree by MAPLE with posterior probability above 95%.

We counted the number of occurrences of single-nucleotide substitutions and their number of descendants in the tree. We then did the same calculations for all pairs of single-nucleotide substitutions found on the same branch in the tree, even those occurring in different parts of the genome in order to allow for the possibility of a mechanism creating simultaneous distant substitutions. Due to the short branches in the SARS-CoV-2 phylogenetic tree, a manageable number of such pairs was observed. When recurrent clusters of >2 nucleotide substitutions were found, these were also tracked (and occurrences of larger clusters were not counted towards the numbers of occurrences of smaller nested clusters).

We identified all genomes descending from any occurrence of the most recurrent mnSs (those in Table 1) on a terminal branch of the tree. For any of these genomes that were part of a cherry (a 2-tip subtree) in the phylogenetic tree, we analyzed sequencing read data (as represented in Viridian quality control files) for both members of the cherry. We wrote a custom python script to extract information about coverage, indels, and variants near the considered mnSs in these genomes.

The phylogenetic distribution of recurrent mnSs was visually investigated using Taxonium v2.0.115 (Sanderson 2022).

We used the program Palindrome in EMBOSS (Rice et al. 2000; minimum palindrome length 15, maximum 25, maximum gap 10, and maximum 4 mismatches) to investigate if inverted repeats might have caused any recurrent MNMs via sequence-directed mutagenesis (Dutra and Lovett 2006; Ladoukakis and Eyre-Walker 2007), but found none overlapping with the recurrent MNMs of Table 1.

The output of RIVET was analyzed with a custom python script to extract putative recombination events informed by the mnSs in Table 1.

We used two tests to quantify the possibility that mnSs might be caused by combinations of individual nucleotide mutations occurring by chance on the same branch. The first is the hypergeometric test: we calculate the probability of observing at least the recorded number of co-occurrences of individual substitutions composing each considered recurrent mnS, assuming that individual nucleotide substitutions are distributed uniformly and independently along the branches of the phylogenetic tree. The test is analogous to the test of association of a set of genes to a specific GO category and is equivalent to Fisher’s exact test (Rivals et al. 2006). As we consider 2,072,111 genomes our rooted tree has 4,144,220 branches, which gives the total number of objects of the hypergeometric distribution. We used the hypergeom.sf hypergeometric survival function in SciPy (Virtanen et al. 2020) for accurate probability calculations. Further, since we consider all possible combinations of mutations, to account for multiple testing we use a Bonferroni correction with total number of tests $(3L{)}^{2}$, where L=29,903 is the genome length and hence 3L the number of possible mutations from the reference genome. For mnSs composed of more than two nucleotide substitutions, we use the conservative procedure of considering only the two rarest nucleotide substitutions.

The hypergeometric test uses the simplifying assumption that all branches are equally likely to contain a certain mutation—this is not however the case in practice, since branches have different lengths; this might make the hypergeometric test liberal. To provide further confidence, we also ran a permutation test: we randomly shuffle all the mutations on the tree, keeping both the number of mutations on each branch and the number of occurrences of each individual nucleotide substitution the same as in the original tree. (In our shuffling, as an approximation, we do not enforce consistency of the mutational history: for example, the same mutation is allowed to be placed more than once on the same branch.)

To evaluate the significance of the association between recurrent MNMs and consensus TRS motifs, we use a binomial test (as implemented in the binomtest function in SciPy; Virtanen et al. 2020) assuming as null hypothesis that MNMs can occur uniformly randomly along the 29,903 bp SARS-COV-2 genome, and testing the significance that 6 out of 15 recurrent MNMs fall within 1–5 bp $5^{\prime }$ of a consensus TRS motif. We conservatively assume 10 consensus TRS motifs along the genome (the 9 in the reference plus one created by mnS3 although this is mostly only present in Alpha and Omicron genomes: see Fig. 4), giving a probability of success of (5×10)/29,903=0.00167 for the null binomial distribution.

To test whether candidate recurrent MNMs might be caused by selection, we investigate their possible enrichment in nonsynonymous mutations which would suggest they might in fact be mnSs composed of distinct 1-nucleotide (1-nt) mutations frequently co-occurring due to selective phenomena like epistasis and compensation. To test this we use a Monte Carlo approach and focus on MNMs involving 2-nt substitutions and no indels as being those most plausibly explained by selection. We assume as null hypothesis that recurrent 2-nt mnSs are only caused by recurrent 2-nt MNMs (not by selection, e.g. compensatory mutations or strong epistatic interactions) and that selection does not prevent recurrent MNMs from being observed (e.g. by causing a strongly deleterious nonsynonymous substitutions) except for nonsense mutations (i.e. early amino acid sequence terminations are assumed lethal). We simulated random pairs of mutations along the ORF1a reference sequence (13,203 bp) as representative of coding sequences in SARS-CoV-2. These pairs sampled all locations and replacement nucleotides with equal probability, with the two mutations 1 bp, 2 bp, and 3 bp apart (as seen in the 2-nt recurrent MNMs in Table 1). In each case we simulated 10,000,000 random mutation pairs, discarding those causing stop codons. We then compared the proportion of different types of substitutions in our simulations with those in the real data. The first statistic we consider is the proportion of 2-nt substitutions composed exclusively of nonsynonymous substitutions. The recurrence of 2-nt mnSs composed of nonsynonymous substitutions might be more easily justified with compensatory or epistatic selective dynamics (our assumption is that selective mechanisms would not typically cause the recurrence of synonymous substitutions), so we hypothesize that, if selection was responsible for a substantial proportion of our recurrent MNMs, there would be an enrichment of 2-nt substitutions composed exclusively of nonsynonymous substitutions among our MNMs compared to these simulations. To give the maximal opportunity for alternative explanations (including selection) to overturn our null hypothesis, we consider a 2-nt mnS to be composed of nonsynonymous substitutions even if both substitutions affect the same codon and the second substitution reverts the amino change caused by the first substitution (as would be for example the case if MNM2 was caused by two separate 1-nt mutations). In our simulations we observe respectively 51.8% (for the 1 bp separation), 52.7% (2 bp separation), and 67.6% (3 bp separation) of mutation pairs composed of two nonsynonymous substitutions (all others involved at least one synonymous substitution). Averaging these three cases based on the proportions observed from our 7 recurrent indel-free 2-nt MNMs (5/7, 1/7 and 1/7 for 1, 2 and 3 bp separation, respectively; see Section “Selection”), we obtain an overall null expected proportion of 54.2% of mutation pairs composed only of nonsynonymous substitutions. We compare the observed proportion in real data against this null expected proportion using a one-sided binomial test. We used a similar approach to test for enrichment in the total number of nonynonymous substitutions within 2-nt mnSs (simulated proportion 76.0%.)

## Supplementary Material

msaf272_Supplementary_Data

## Data Availability

The alignment, metadata, and inferred tree are publicly available on Zenodo (De Maio n.d.). The list of recombination events inferred by RIVET, and the list of cherry samples analyzed here, are available from https://github.com/NicolaDM/MAPLE/tree/main/multinucleotideMutations.
