# Supplementary material for: Highly Recurrent Multinucleotide Mutations in SARS-CoV-2
Source: Mol Biol Evol. 2025 Oct 24;42(11):msaf272. doi: 10.1093/molbev/msaf272 (PMC12619124; doi:10.1093/molbev/msaf272)
Supplement: msaf272_Supplementary_Data [file msaf272_supplementary_data.zip › File_S1.pdf]

Highly recurrent multi-nucleotide mutations in SARS-CoV-2 – Supplementary File S1

Assessment of recurrent multi-nucleotide mutations via read alignment visualization

Here we visualise read alignments of several blindly selected "cherry" samples containing the recurrent multi-nucleotide mutations (MNMs) discussed in the main text. The read data was retrieved from the [EBI API](https://www.ebi.ac.uk/ena/portal/api/) (https://www.ebi.ac.uk/ena/portal/api/) and was processed using minimap2 [1] and samtools [2] with the following pipeline.

Command used to map reads to the reference NC_045512.2 (https://www.genome.jp/dbget-bin/www_bget?refseq:NC_045512 the same used by Viridian [3]):
*minimap2 -t 8 -a -x sr ref_path read_path_1 read_path_2 | *

To add mate-pair information:
*samtools fixmate -u -m - - | *

To sort the alignment:
*samtools sort -T /tmp/sorting -@4 - | *

To flag duplicates and create a BAM file:
samtools markdup -@8 --reference ref_path bam_path

To generate a BAI file:
samtools index bam_path

We visualized the aligned reads in IGV [4], from which we present screenshots below. We chose blindly "cherry" samples for each recurrent MNM in the main text for visualization, and we include below a screenshot for each such sample – no selection was made of which samples to include in this document. First, we picked two samples from each recurrent MNM, and in cases where at least one sample for the considered MNM had any issues, we picked a third one.

The approach we use here for processing reads is different from the one used by Viridian (which we use in the main text). Viridian infers primers from the read data, and trims the corresponding positions of the reads, which we do not do here. Also, the approach we use here seems to trim read ends more aggressively in the presence of MNMs, suggesting a

stricter cut-off with respect to the number of substitutions allowed at read ends. Differences between the two approaches (the Viridian one used for the main text and the minimap2 approach used here) can be useful to highlight issues specific to either of the two approaches.

Each screenshot shows aligned reads hundreds of bp around the considered MNM. Reads are colored based on their strand orientation (red reads are 5′ to 3′, blue reads 3′ to 5′). SNPs relative to the reference are colored with the following scheme: A: green, T: red, C: blue, G: orange. In the top panel, black bars show the total depth at each position. Black arrows in the plots point to the location of the considered MNMs.

The main purpose of these figures is to show that recurrent MNMs are supported by the central section of the reads (excluding possible artefacts caused by sgRNA or primers), and ideally are supported by reads from both orientations.
Furthermore, recurrent MNMs typically have sufficiently high depth and homozygosity to exclude artefacts caused by contamination, amplicon drop-out, and other sequencing and alignment issues.

All samples described and analysed here have been processed and shared in [3].

Table of the visualized samples

ID	MNM	MNM-containing samples	Samples without MNMs in same cherries
MNM1	C21302T-C21304A-G21305A	ERR7465684, SRR15753566, ERR6759625	ERR7487407, SRR15748149, ERR7208354
MNM1a	C21304A-G21305A	ERR7377380, SRR21372376, ERR6755120	ERR7010071, SRR21451753, ERR6765491
MNM2	A28877T-G28878C	ERR5176732, SRR21041378, SRR21582970	ERR5277758, SRR19919918, SRR20467128
MNM3	G27382C-A27383T-T27384C	SRR18236038, ERR6892550, ERR6610710	SRR21347599, ERR6752751, ERR6520822
MNM4	T26491C-A26492T-T26497C	ERR6596507, ERR5178990, ERR6328373	ERR7004352, ERR5178220, ERR6443701
MNM5	G27758A-T27760A	ERR7312482, ERR5399348, SRR19297838	ERR7308601, ERR5279147, SRR20935110
MNM6	C25162A-C25163A	ERR5549510, SRR20920049, ERR6608959	ERR5531769, SRR22291448, ERR6295314
MNM7	T27875C-C27881T-G27882C-C27883T	ERR7035845, SRR18098335, ERR8482390	ERR7139582, SRR18052379, ERR7868849
MNM7a	C27881T-G27882C-C27883T	SRR17060468, SRR14428899, SRR20928771	SRR16745827, SRR14330778, SRR20976305
MNM8	T21294A-G21295A-G21296A	SRR20580404, ERR7466894, SRR15587687	SRR19289061, ERR7578505, SRR17448119
MNM9	A27038T-T27039A-C27040A	ERR7152606, SRR18772658, ERR4638176	ERR6664778, SRR20393097, ERR4637647
MNM10	A21550C-A21551T	SRR16252410, ERR5292401, SRR20545163	SRR16326991, ERR5278520, SRR20934637

MNM11	C13423A-C13424A	SRR18737020, ERR6113074, ERR7317567	SRR17365736, ERR6332018, ERR7307921
MNM12	A4576T-T4579A	SRR21008000, SRR15875360, SRR20773344	SRR20637902, SRR19993175, SRR19719604
MNM13	A20284T-T20285C	ERR5316710, ERR6930561, SRR16581479	ERR5317047, ERR6907226, SRR15138232

MNM1 C21302T-C21304A-G21305A:

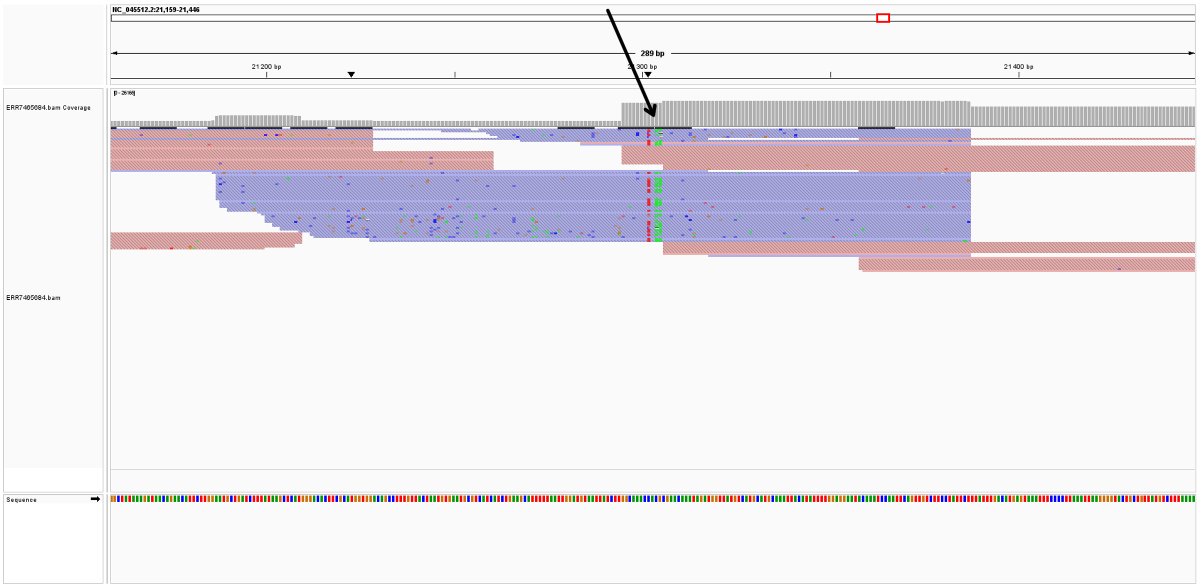

Sample ERR7465684: The MNM appears heterozygous in the sample, with around 20% frequency in the reads, although the actual frequency of the MNM is likely much higher due to the presence of a primer at the MNM positions on the 5′ to 3′ reads, as detected by Viridian. The MNM appears to be missing from the read reads, likely because it was trimmed due to the additional number of substitutions at the read end compared to the red reads without the MNM.

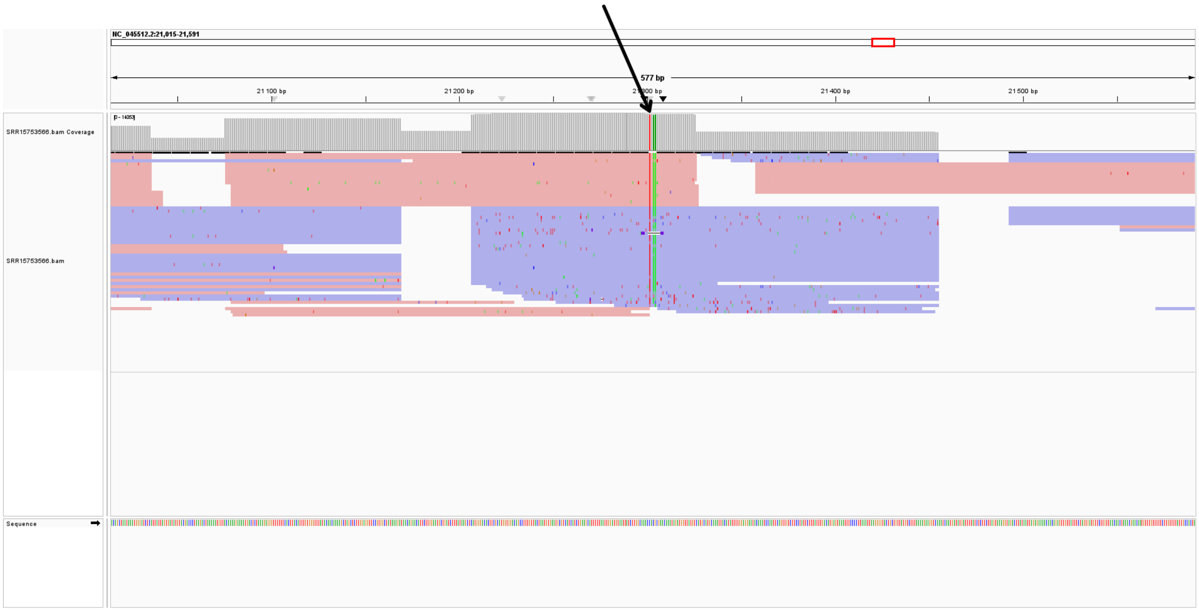

Sample SRR15753566: Nothing odd noted.

MNM1a C21304A-G21305A:

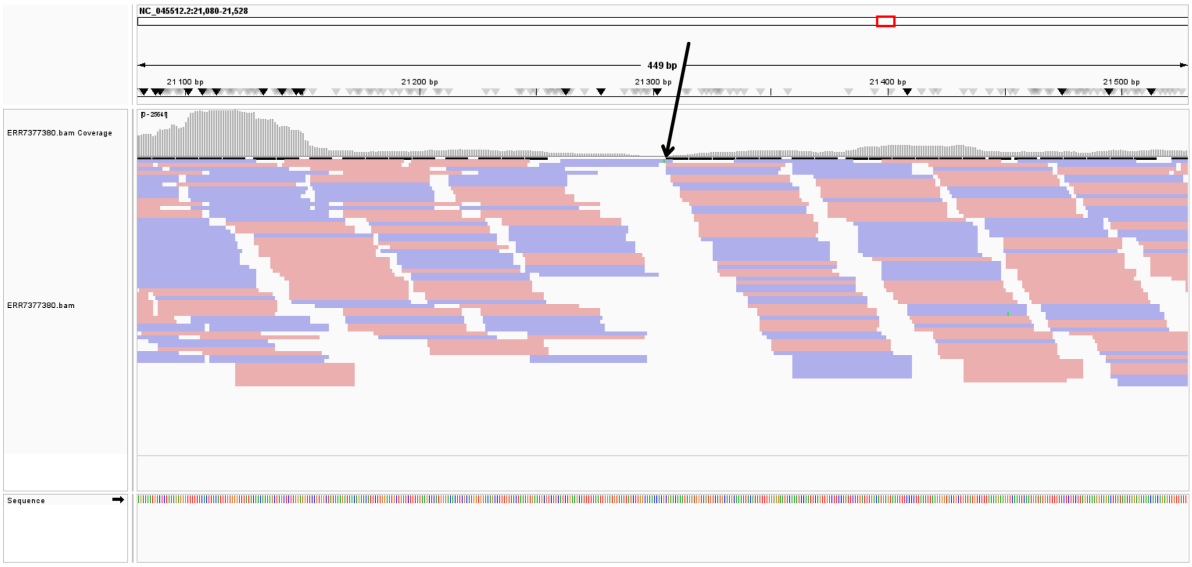

ERR7377380: Large dropout to ~1x near the MNM. This dropout is not observed in the Viridian assembly (considered in the main text), possibly reflecting a stricter read trimming in the approach used here.

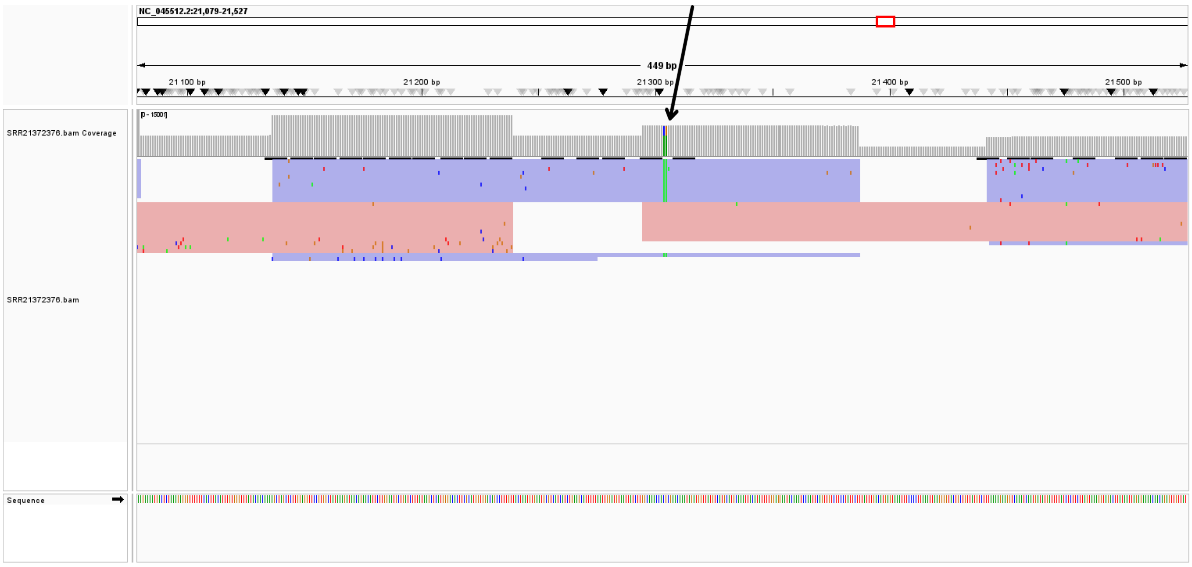

SRR21372376: The MNM is heterozygous at a frequency around 70%. This is not the case for Viridian (used in the main text), which trims the red reads in their overlap with MNM1a due to the presence of an inferred primer.

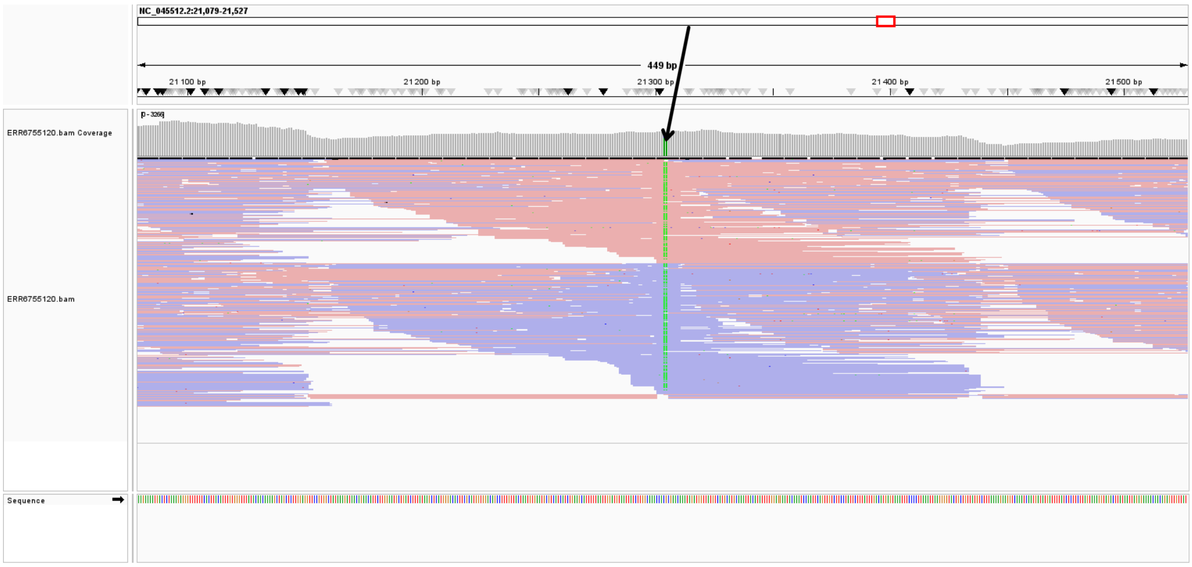

ERR6755120: The MNM is heterozygous at a frequency around 80%. Nothing else odd noted.

MNM2 A28877T-G28878C:

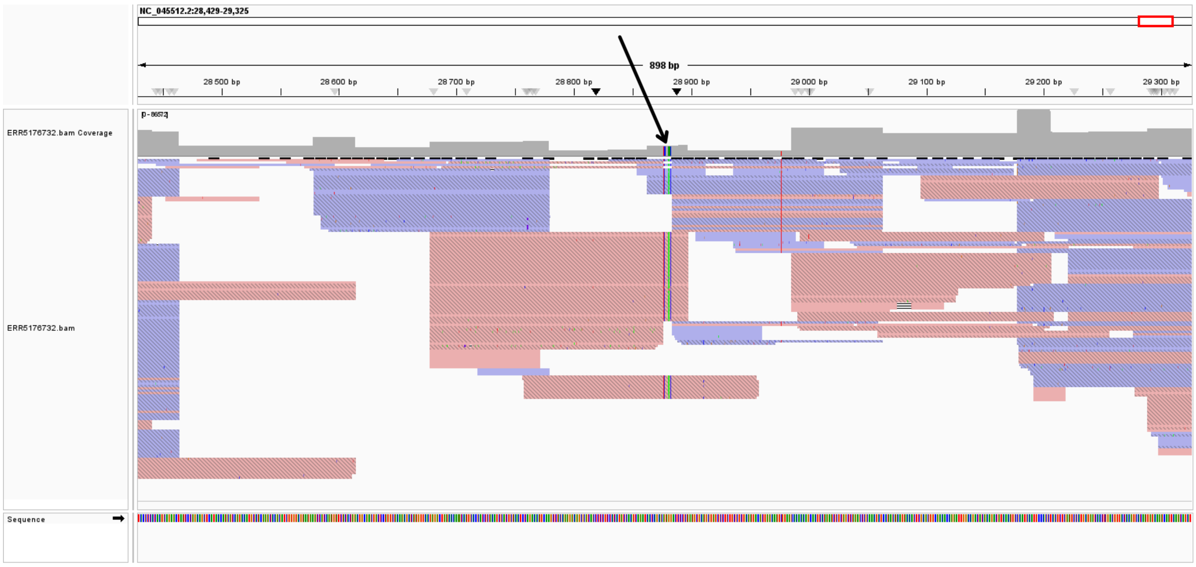

Sample ERR5176732: Nothing odd noted.

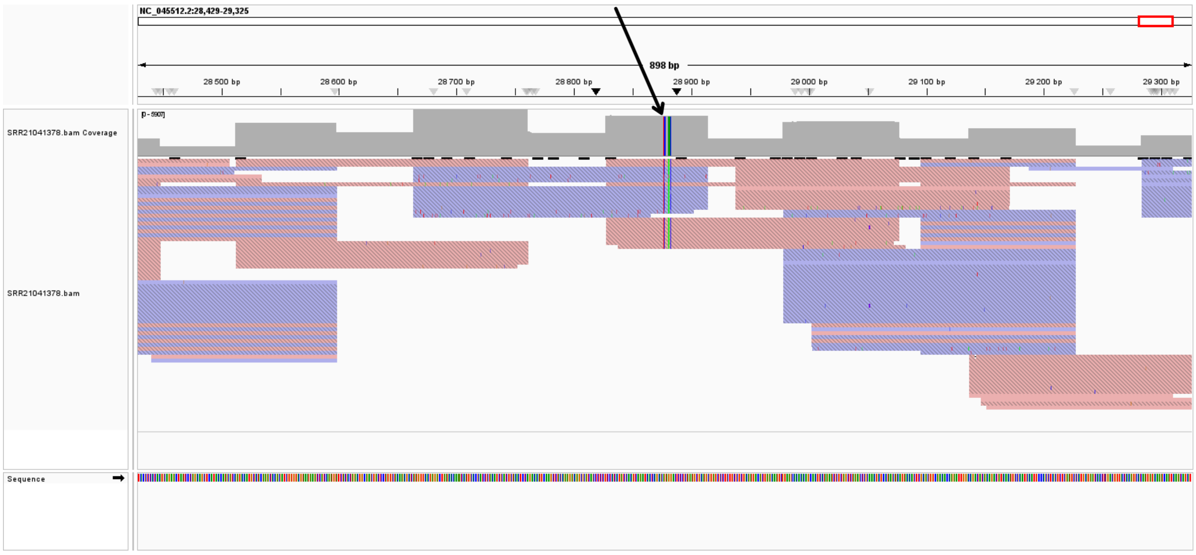

Sample SRR21041378: Nothing odd noted.

MNM3 G27382C-A27383T-T27384C:

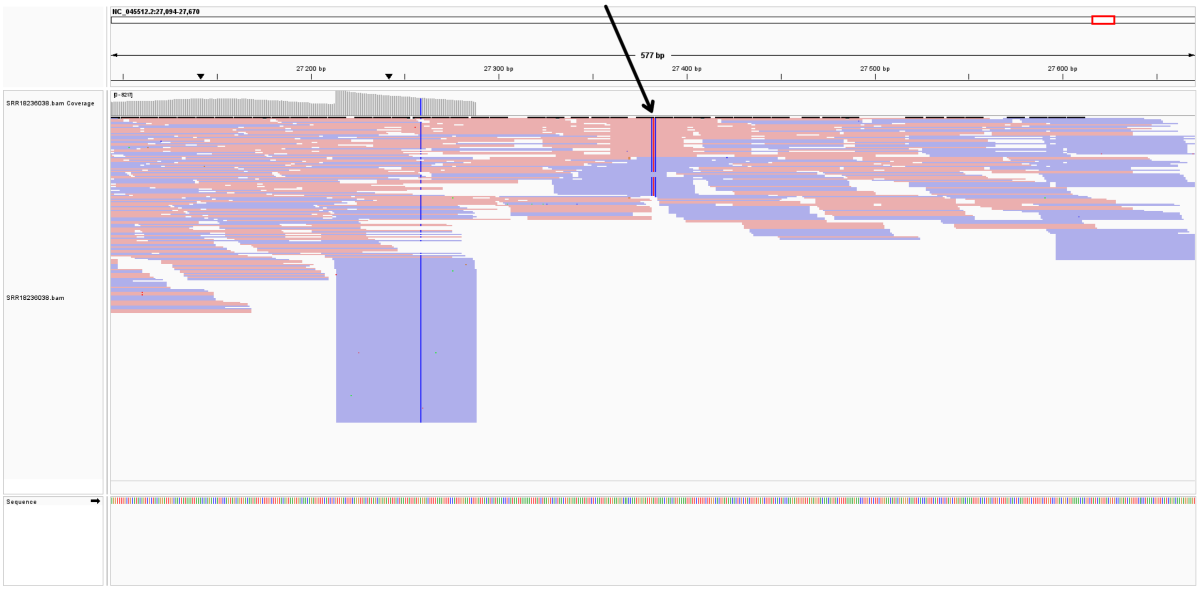

Sample SRR18236038: nothing odd noted, although there is a drop in depth to around 100x near the MNM. The Coverage barplot here shows a maximum of 8217x, but not all the reads mapping to this region are shown, hence why the drop in depth is not reflected in the proportion of mapped reads visualized in the lower part of the plot.

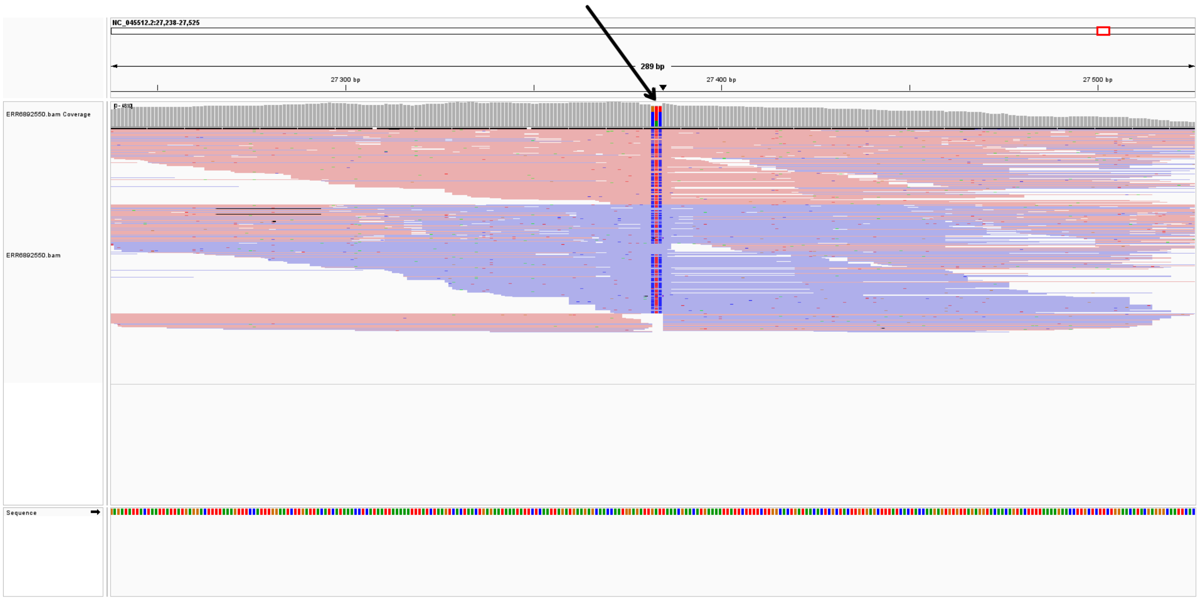

Sample ERR6892550: the MNM is heterozygous at a frequency of around 70%.

MNM4 T26491C-A26492T-T26497C:

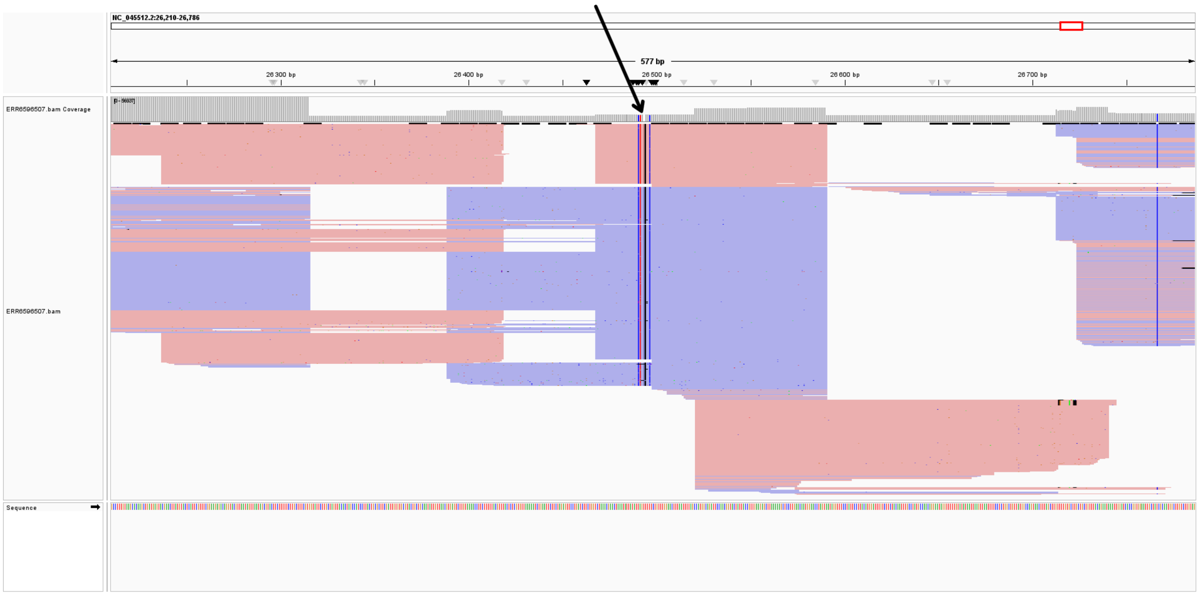

Sample ERR6596507: Nothing odd noted.

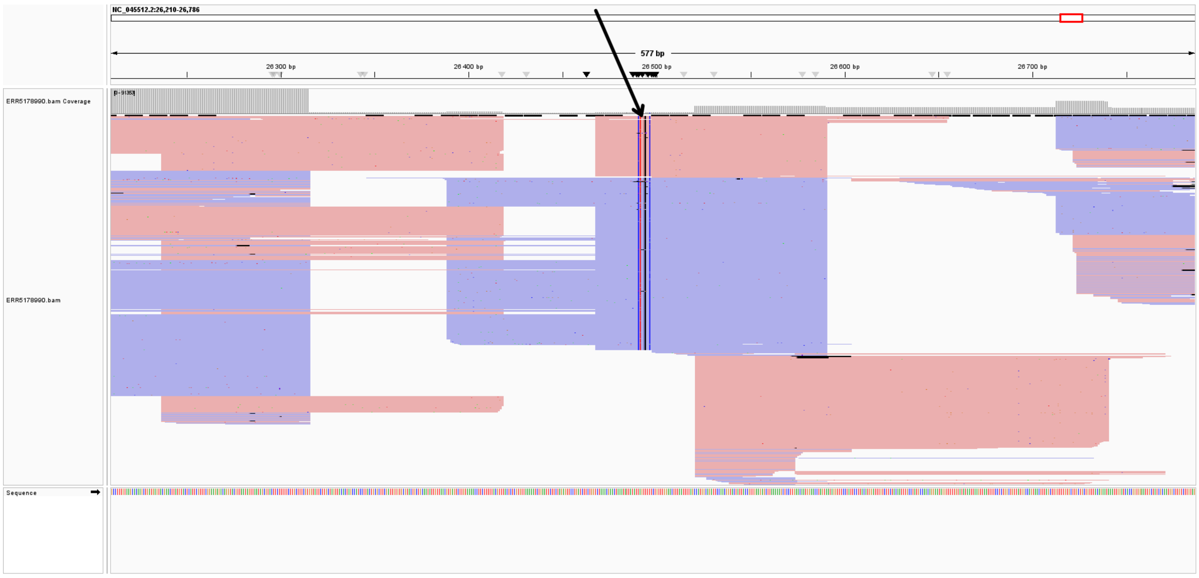

Sample ERR5178990: Nothing odd noted.

MNM5 G27758A-T27760A:

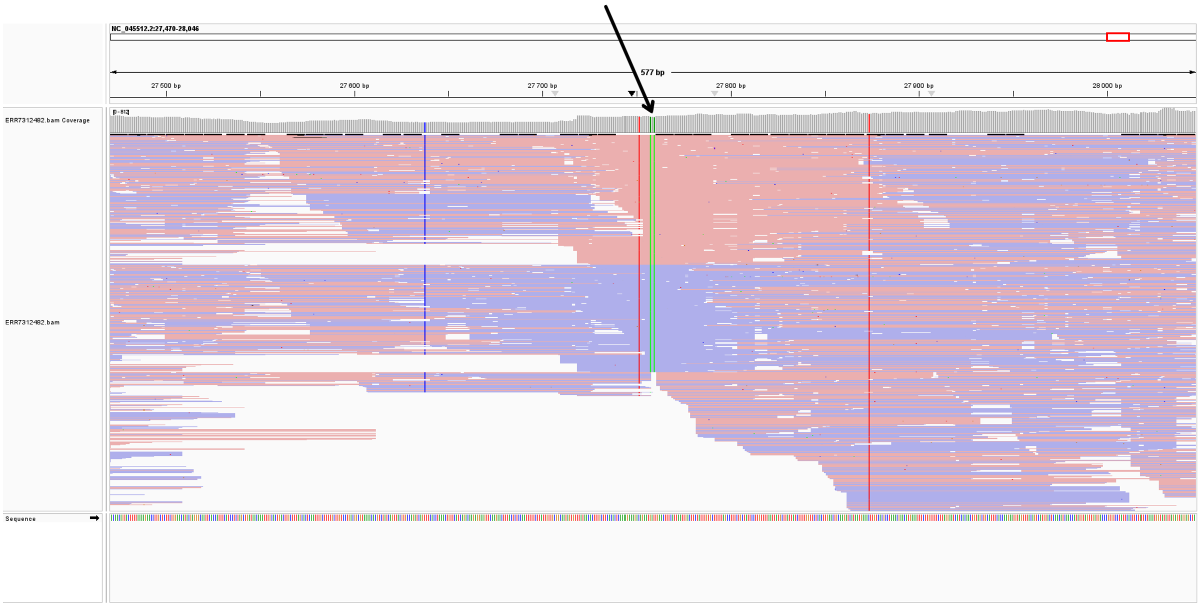

Sample ERR7312482: Nothing odd noted.

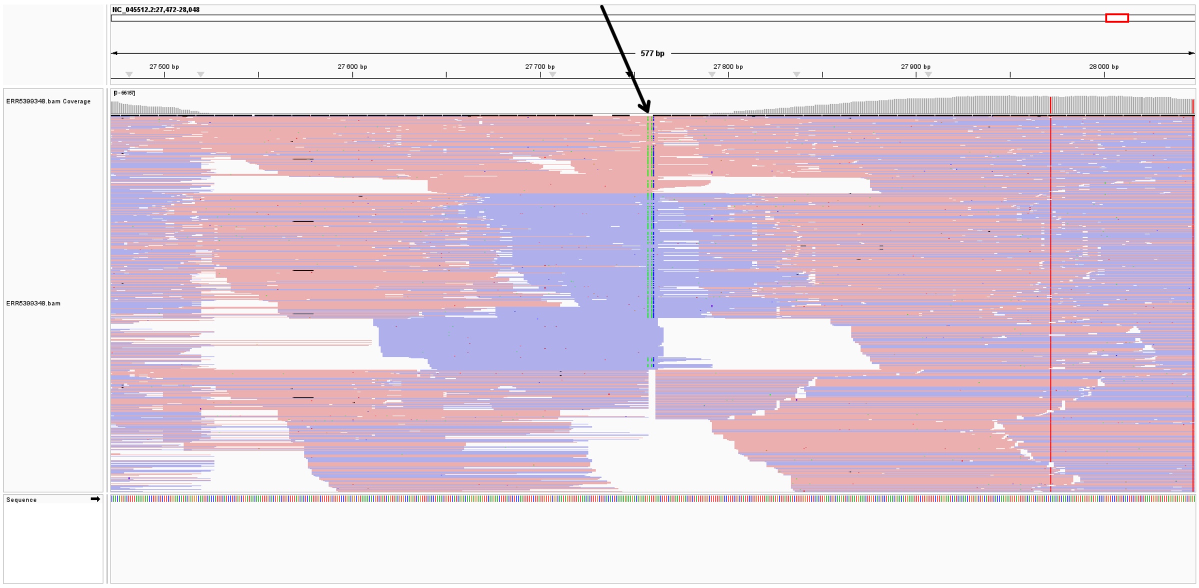

Sample ERR5399348: The MNM is very heterozygous (56 - 44%). In the Viridian read alignment (used for the main text) the frequency of the two variants is quite different, with the MNM5 allele observed in 600x vs 50x reads. This is again likely attributable to different strategies for read end trimming, which result here in many read ends containing MNM5 being trimmed here.

MNM6 C25162A-C25163A:

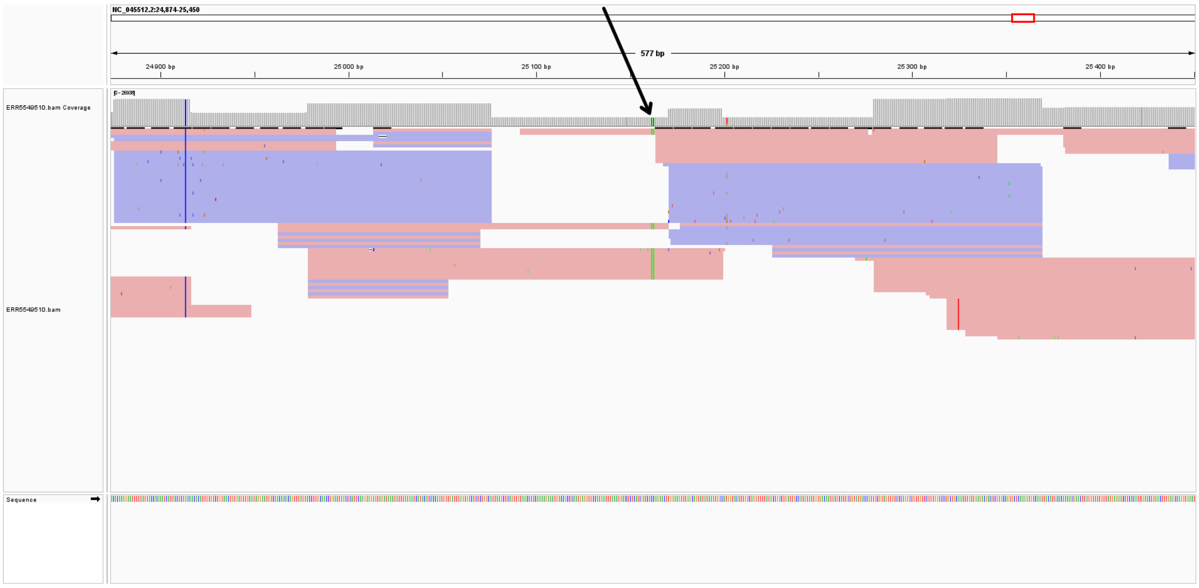

Sample ERR5549510: Nothing odd noted.

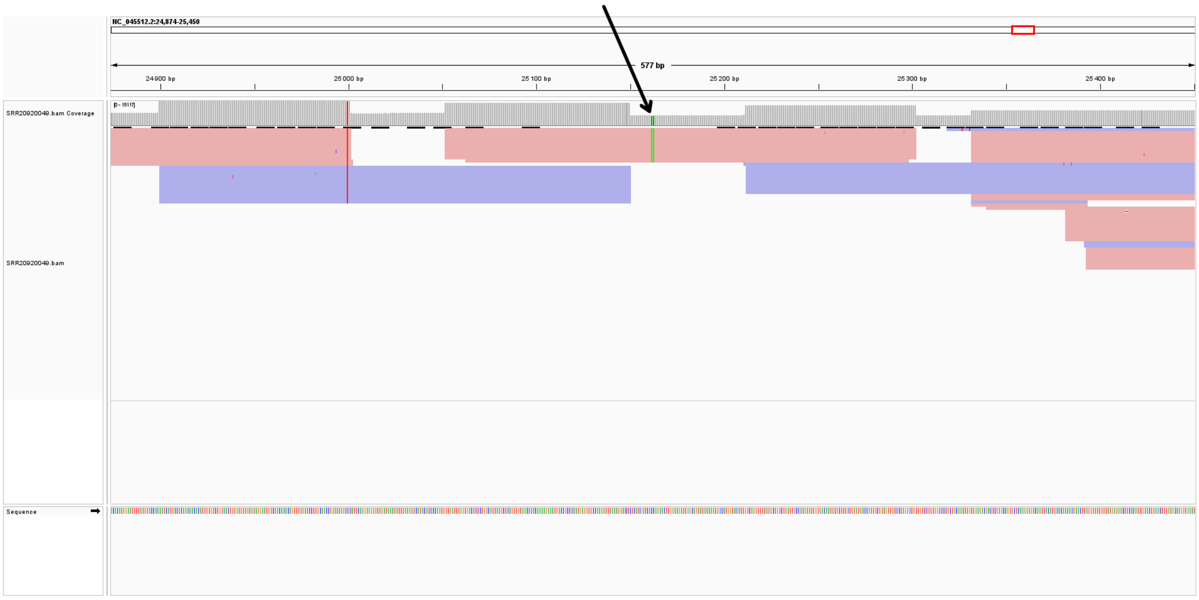

Sample SRR20920049: Nothing odd noted.

MNM7a C27881T-G27882C-C27883T:

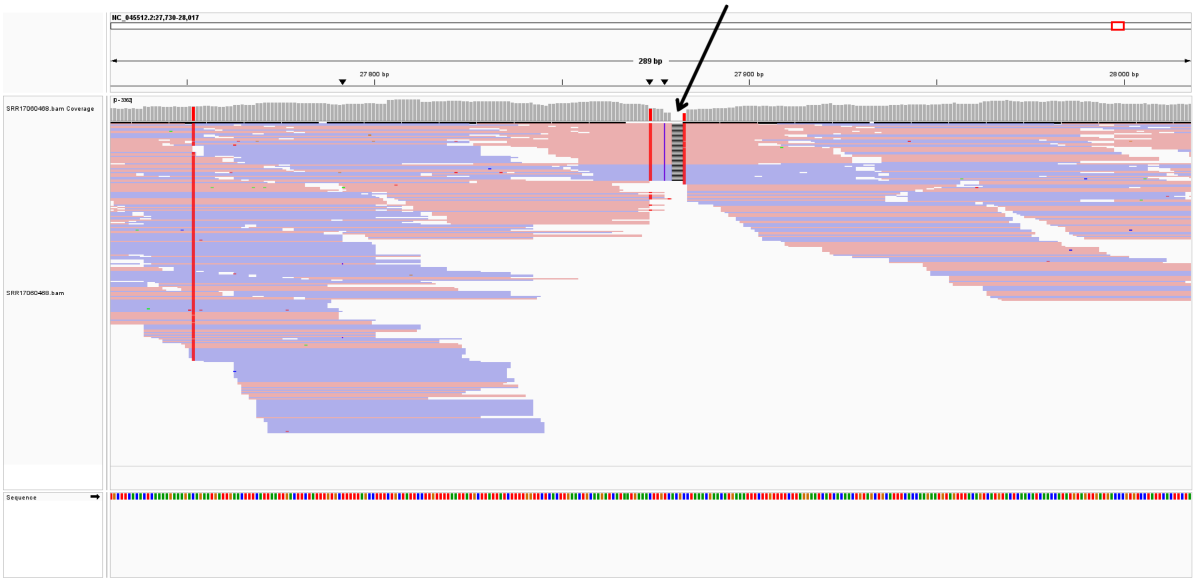

Sample SRR17060468: nothing odd noted. A slight drop in coverage near MNM7 can again be attributed to the trimming of reads containing the MNM near the read end.
A different set of SNPs is annotated here (using minimap2 to align reads to the reference genome) than in our MAFFT [5] alignment of the consensus sequence to the reference genome, but these are just alternative possible alignments to the reference:

Reference:	T T G – T C A C G C C T
minimap2:	T T G T T C – – – T C T
MAFFT:	T T G – T T C T – – C T

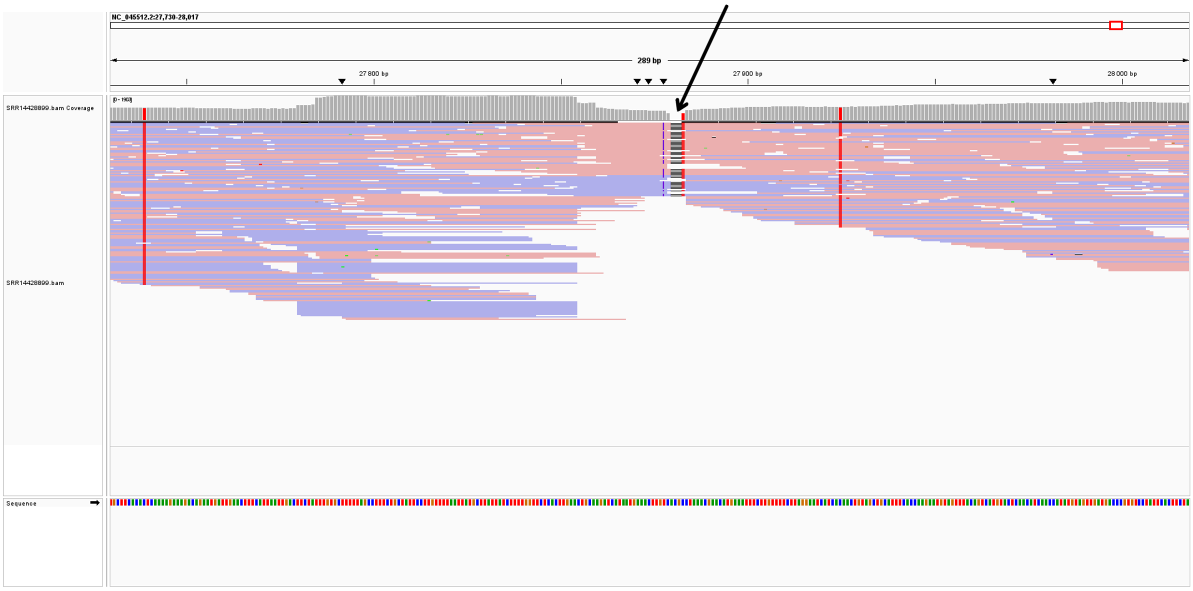

Sample SRR14428899: Nothing odd noted, as for SRR17060468.

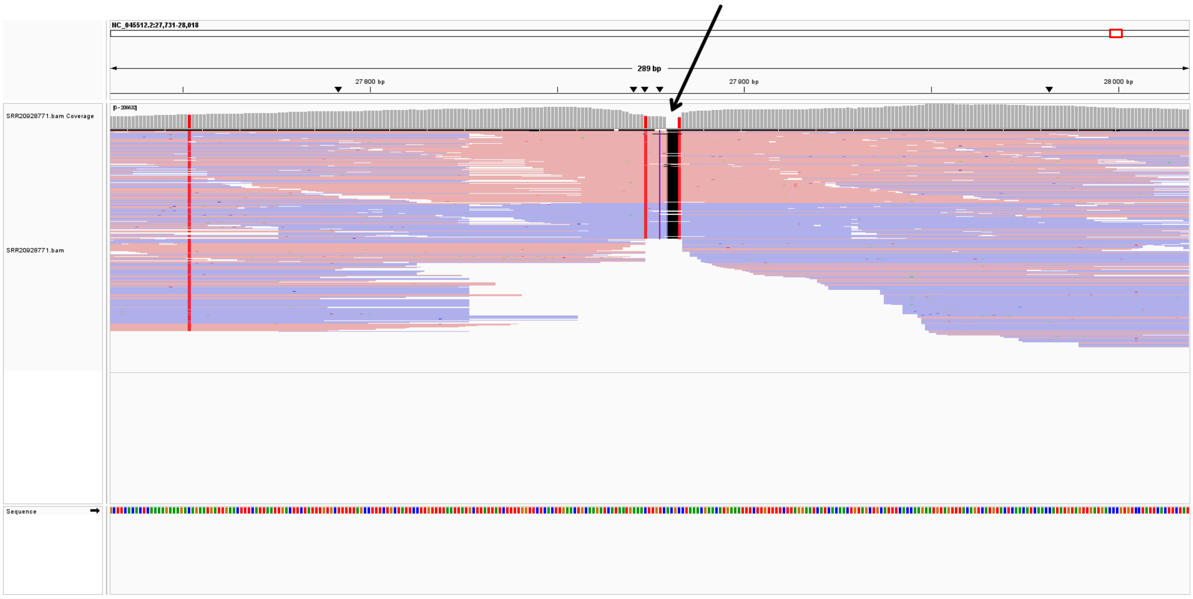

Sample SRR20928771: Nothing odd noted, as for SRR17060468.

MNM7 T27875C-C27881T-G27882C-C27883T:

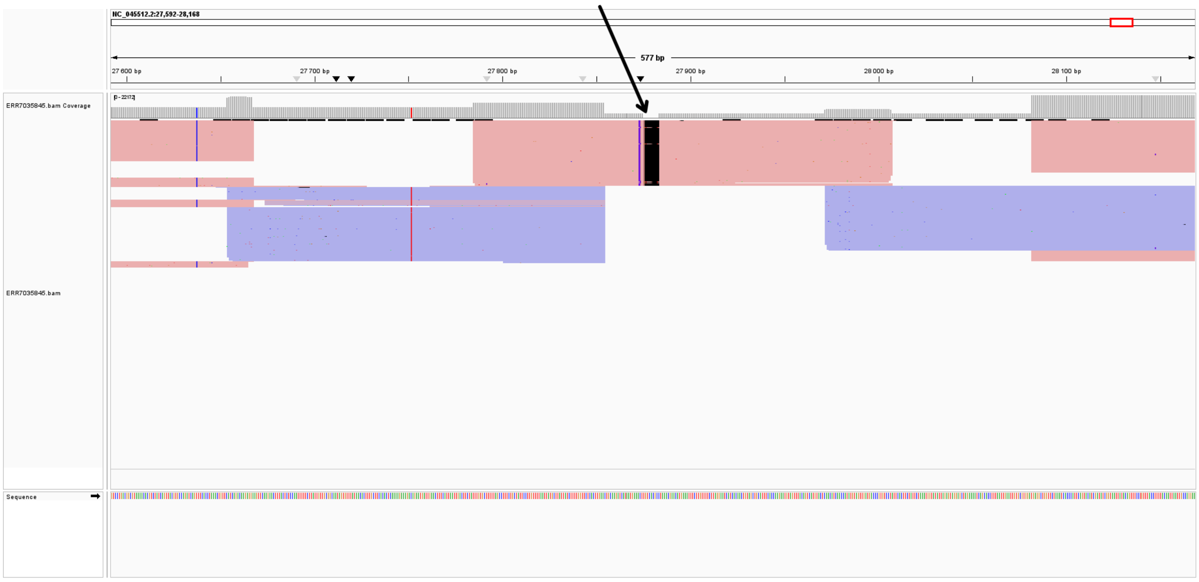

Sample ERR7035845: Nothing odd noted. A different set of SNPs is annotated here, again as a result of different pairwise alignments of the same sequences to the reference by minimap2 (used here) and MAFFT (used in the main text to align the Viridian consensus sequence of the sample to the reference genome):

Reference: A – – – – – – C T T G T C A C G C C T
minimap2: A T C T G T T C T – – – – – – – – – – C T
MAFFT: A – – – – – – T C T G T T C T – – C T

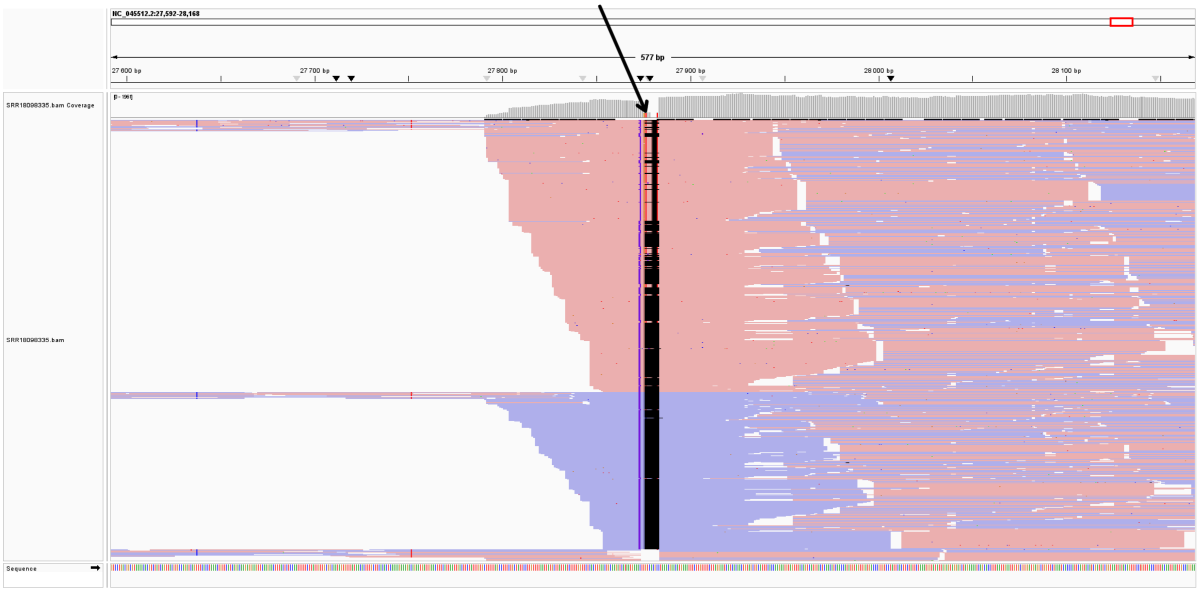

Sample SRR18098335: Nothing odd noted, as in sample ERR7035845.

MNM8 T21294A-G21295A-G21296A:

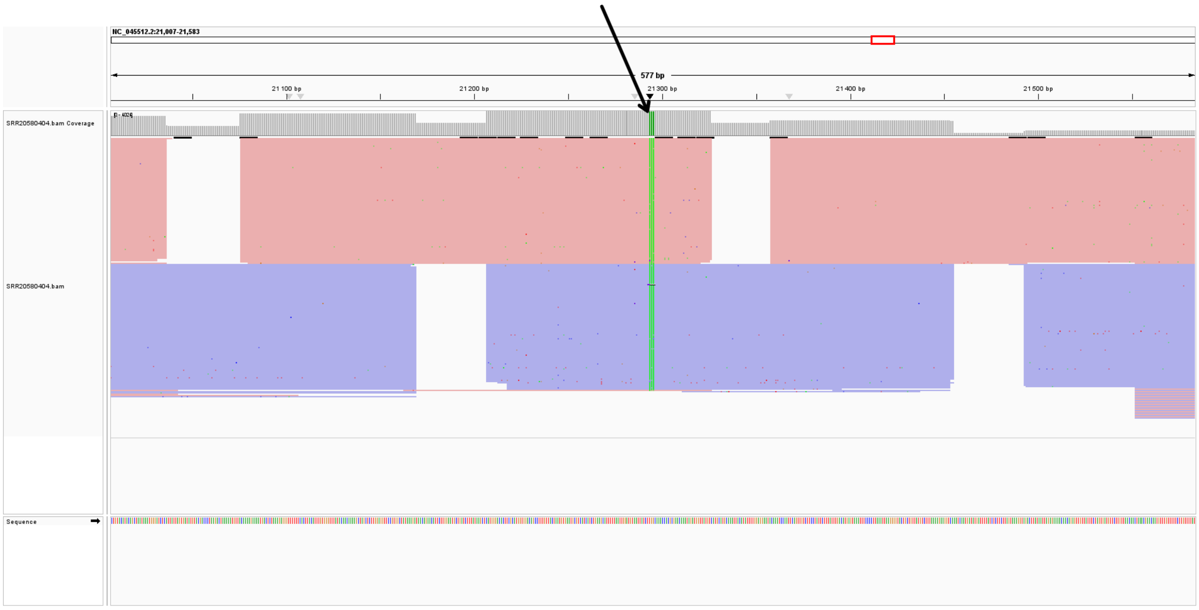

Sample SRR20580404: Nothing odd noted.

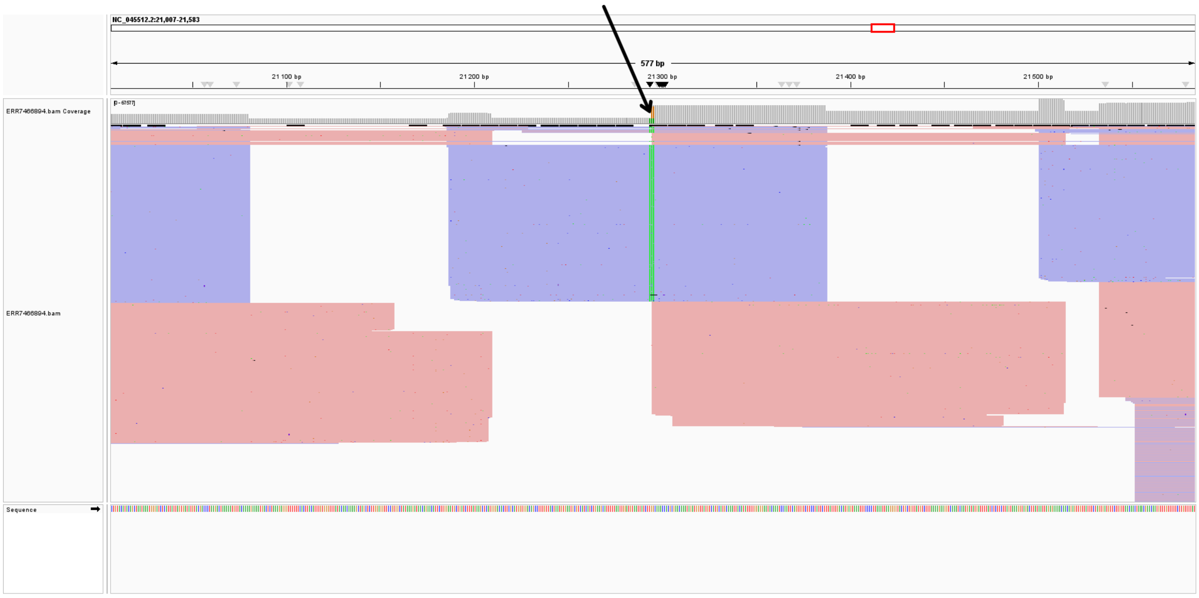

Sample ERR7466894: here part of MNM8 appears heterozygous, but this is not the case for Viridian (used to process the read data in the main text). In fact, Viridian infers a primer for the red reads at the position of MNM8, and consequently trims the red reads in their overlap with MNM8, resulting in a homozygous call for MNM8.

MNM9 A27038T-T27039A-C27040A:

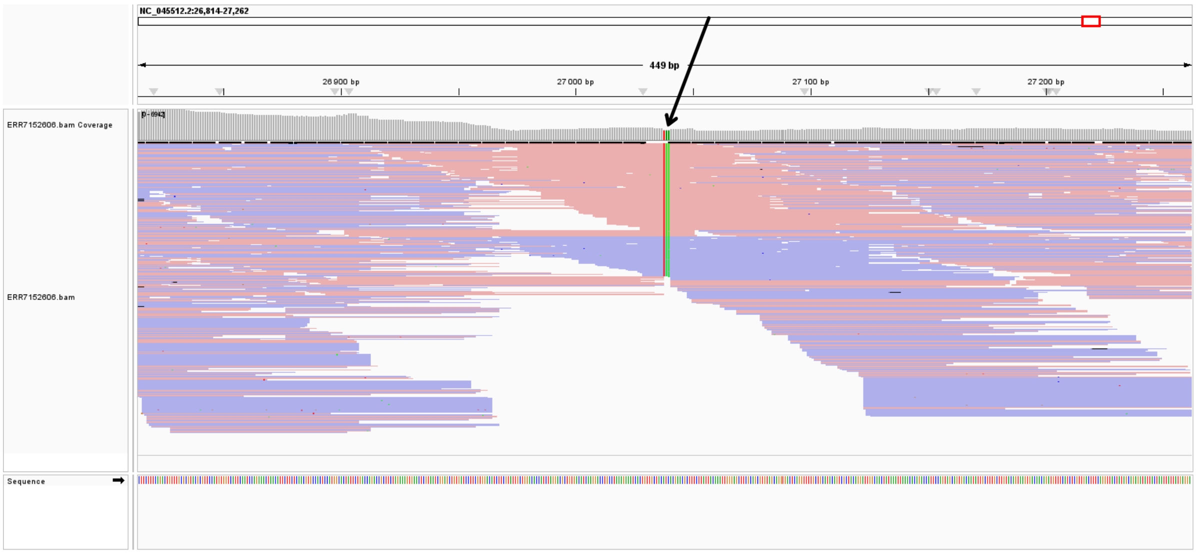

Sample ERR7152606: Nothing odd noted.

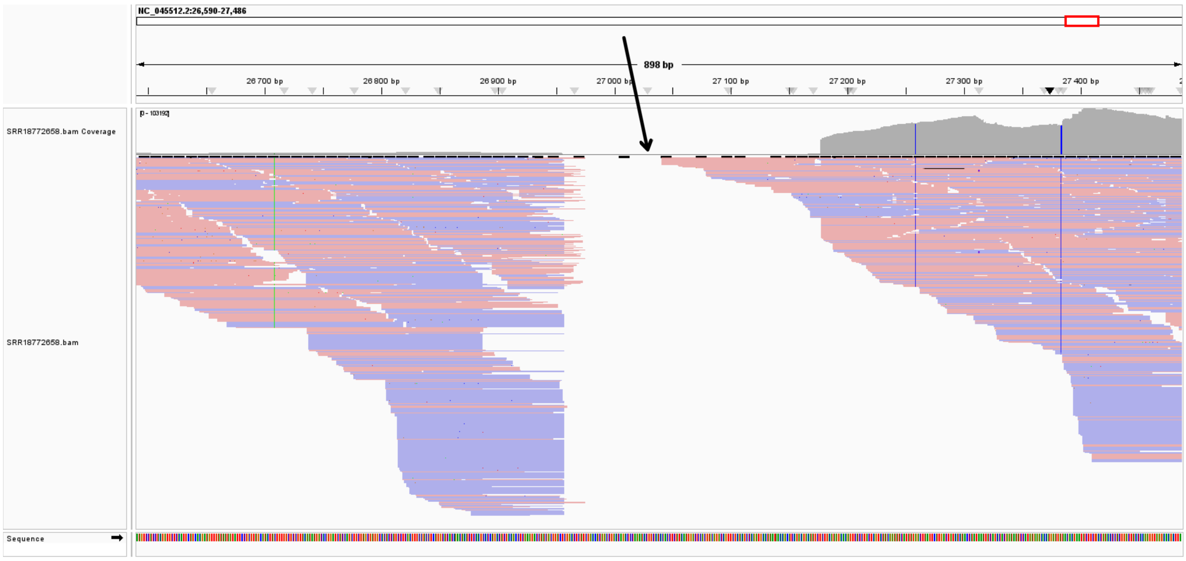

Sample SRR18772658: depth drops near the MNM location from around 27kx to 10x (the coverage barplot on the top of the figure has a linear scale, but, to aid visualization, not all the reads mapping to this region are shown in the lower part of the plot, for which therefore the apparent coverage does not correspond to the actual coverage of the sample). Viridian (used to process the read data for the results of the main text) at the MNM location has higher depth (around 25x), probably because of less stringent read trimming thresholds, and it therefore makes a consensus call for MNM9.

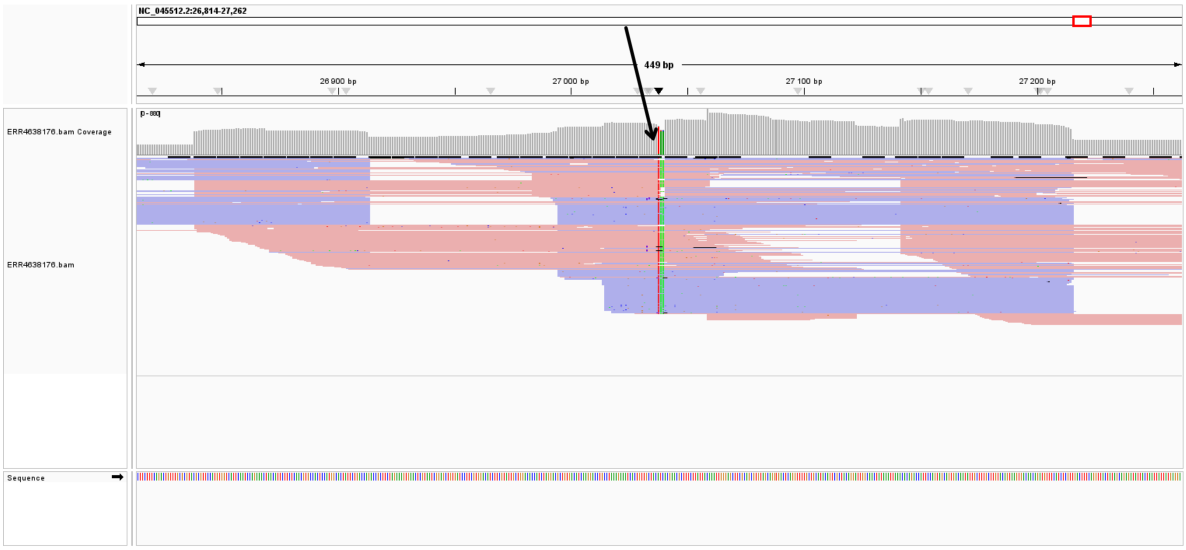

Sample ERR4638176: Nothing odd noted.

MNM10 A21550C-A21551T:

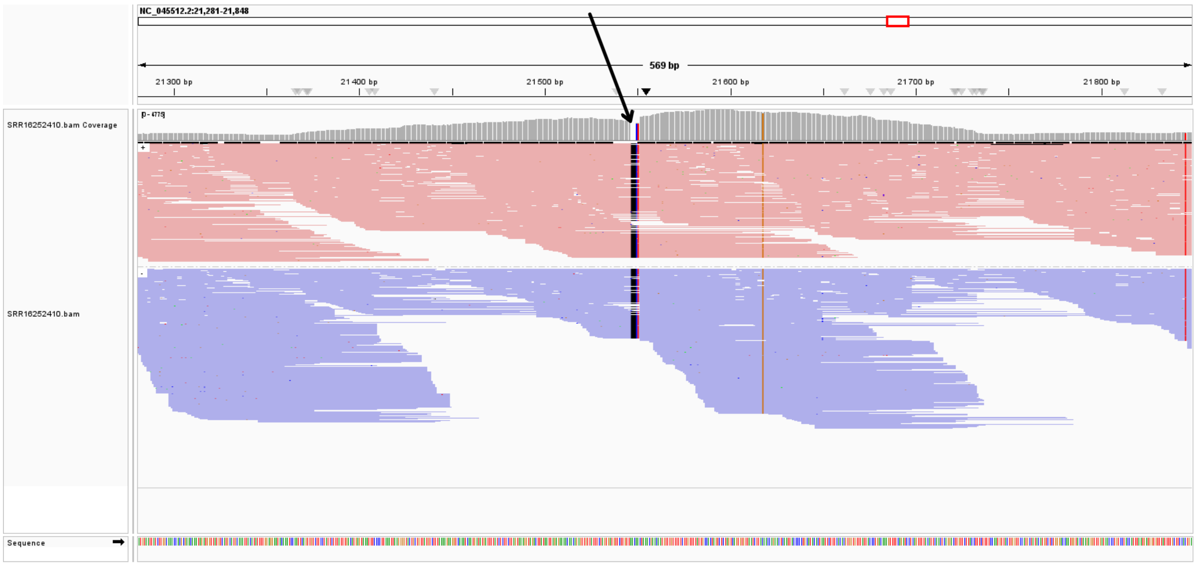

Sample SRR16252410: The deletion at positions 21547-21549 is as reported in Figure 7 in the main text.

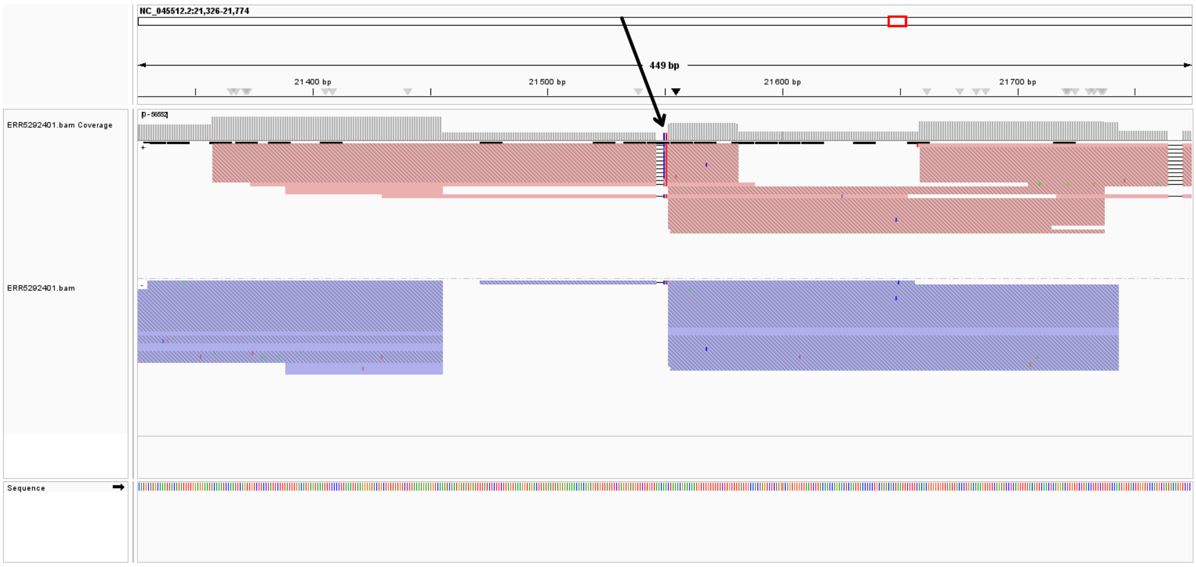

Sample ERR5292401: The MNM is supported by reads from both orientations, but it appears that read trimming causes a sudden drop in depth at the MNM positions.

MNM11 C13423A-C13424A:

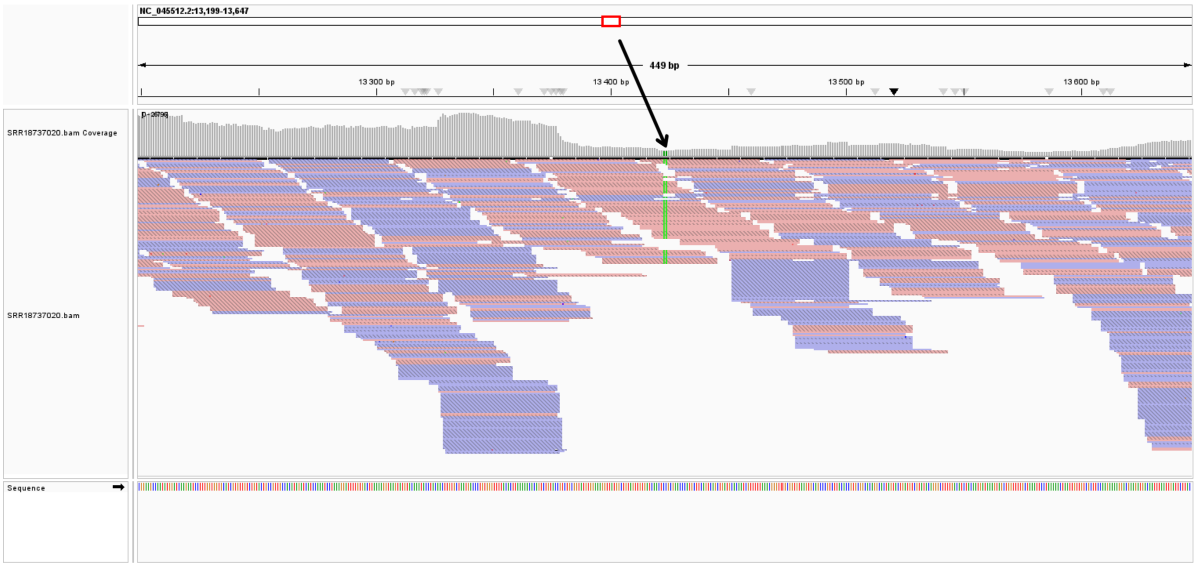

Sample SRR18737020: Nothing odd noted.

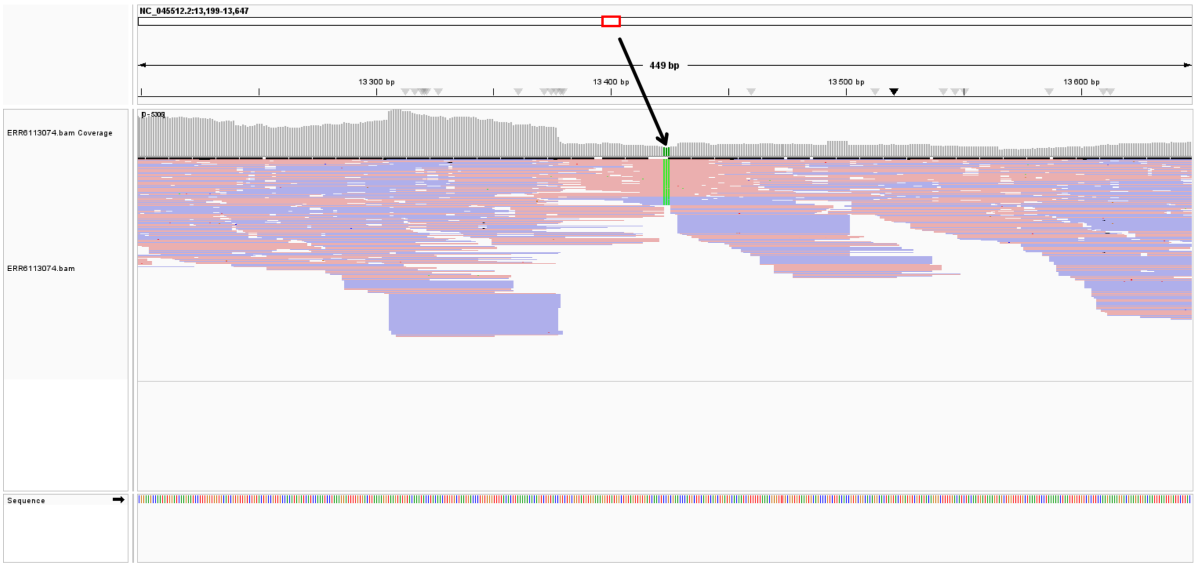

Sample ERR6113074: Nothing odd noted.

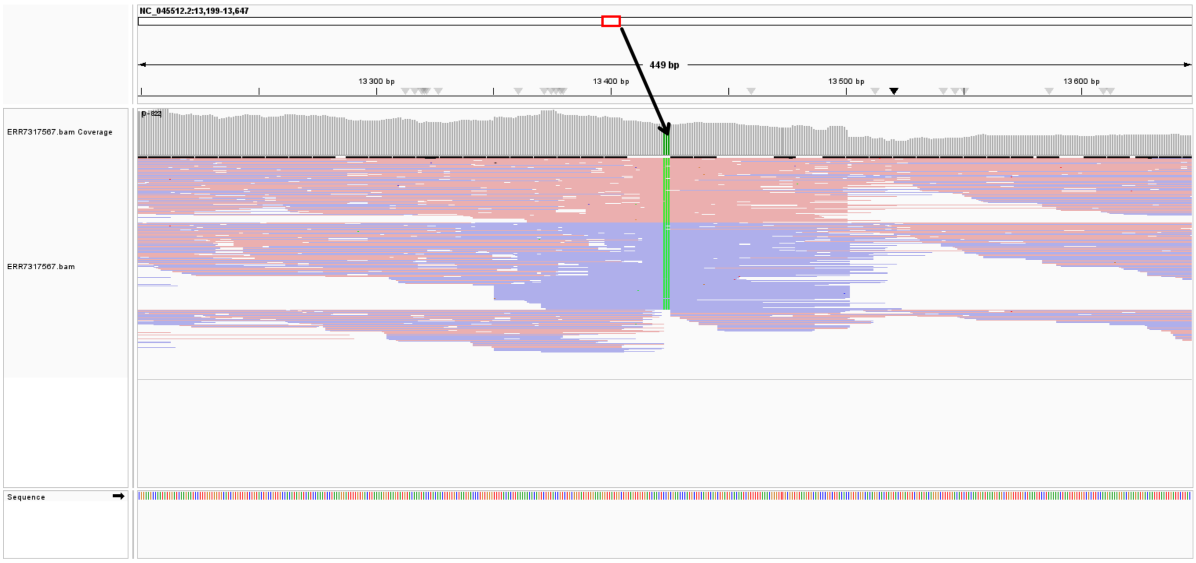

Sample ERR7317567: Nothing odd noted.

MNM12 A4576T-T4579A:

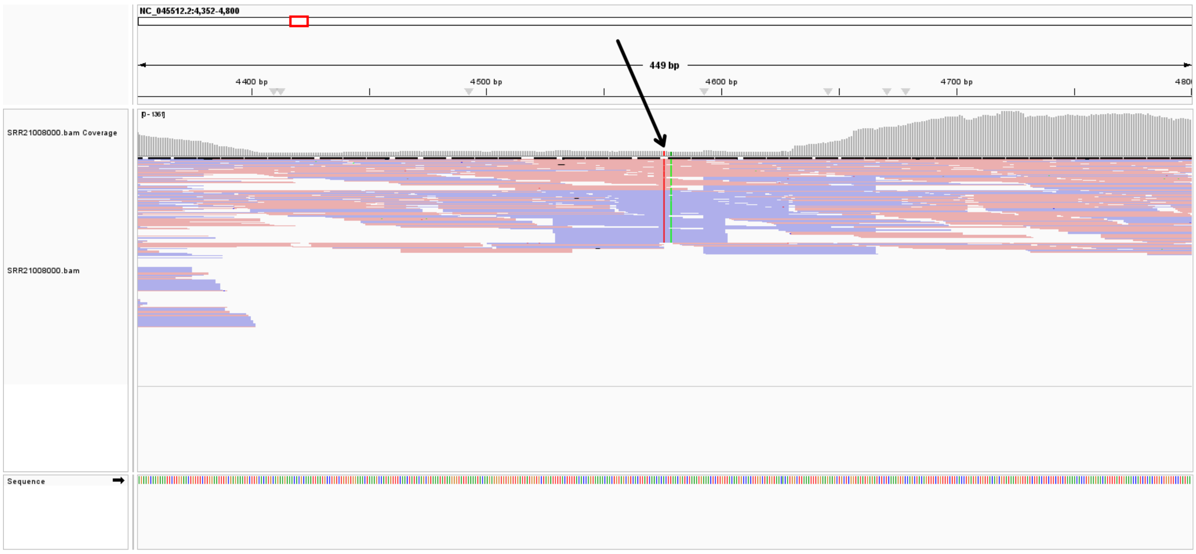

Sample SRR21008000: Relatively low depth around the MNM (150x) compared to nearby regions (maximum depth 1361x within the plot).

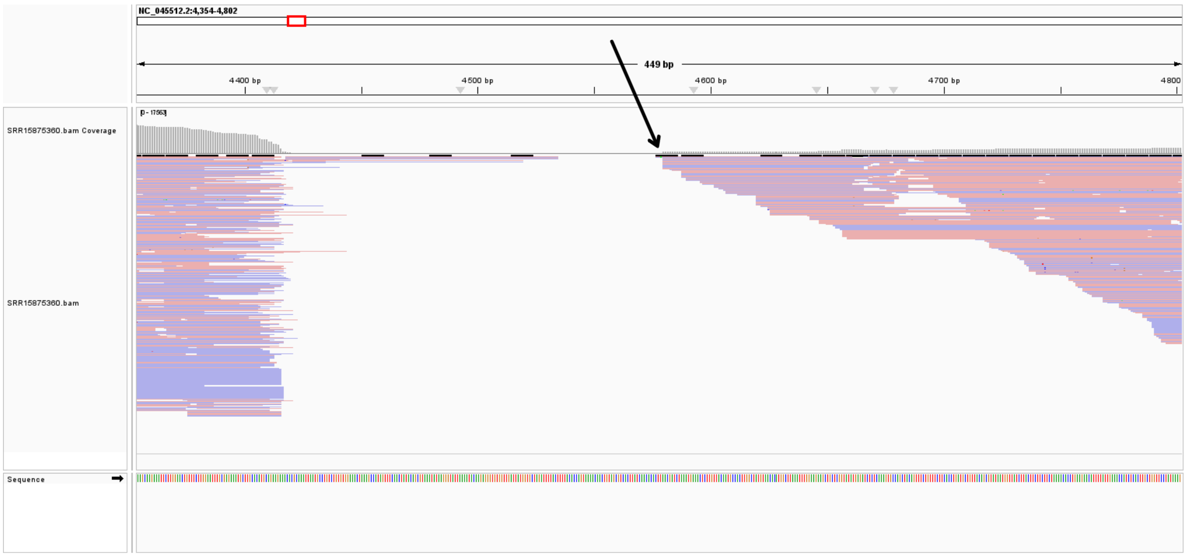

Sample SRR15875360: Very low depth (~10x) near the MNM. Viridian has 600x depth here, suggesting very substantial differences in read trimming.

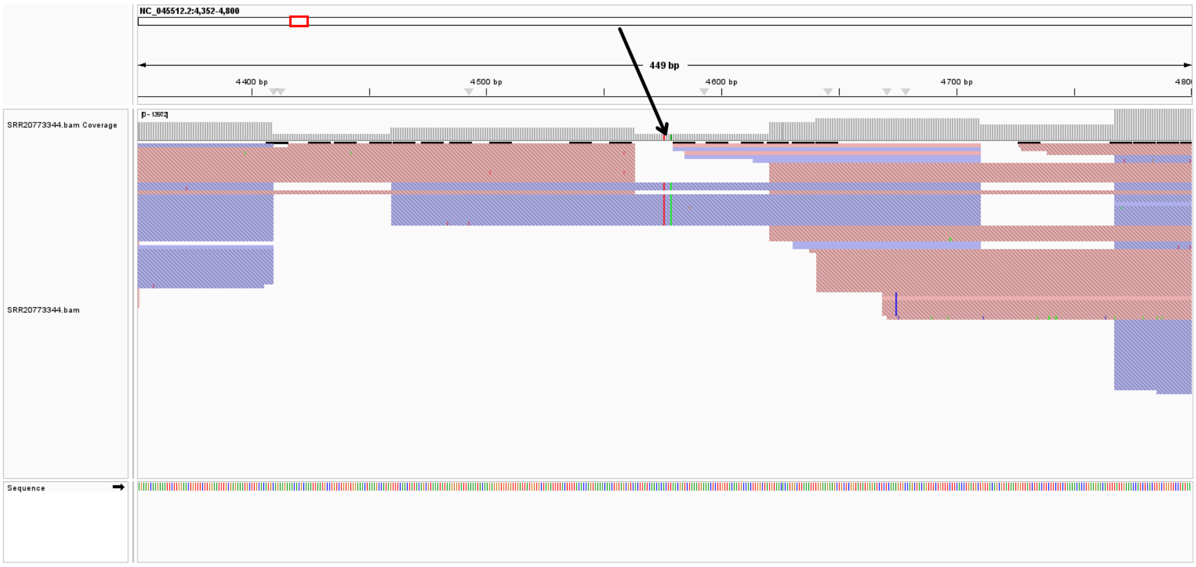

Sample SRR20773344: Nothing odd noted.

MNM13 A20284T-T20285C:

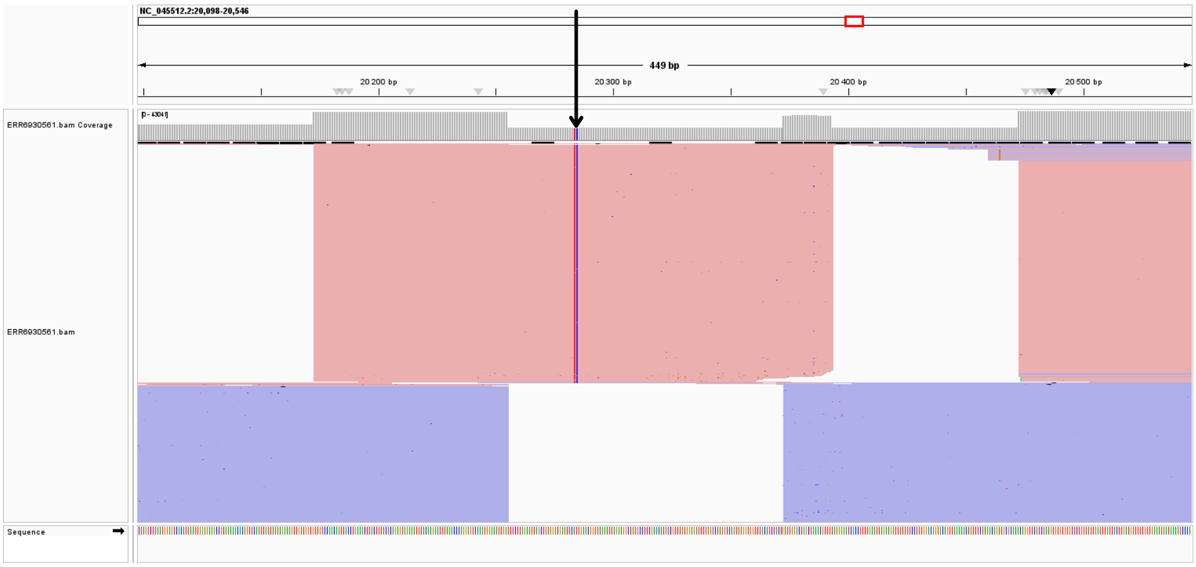

Sample ERR6930561: Nothing odd noted.

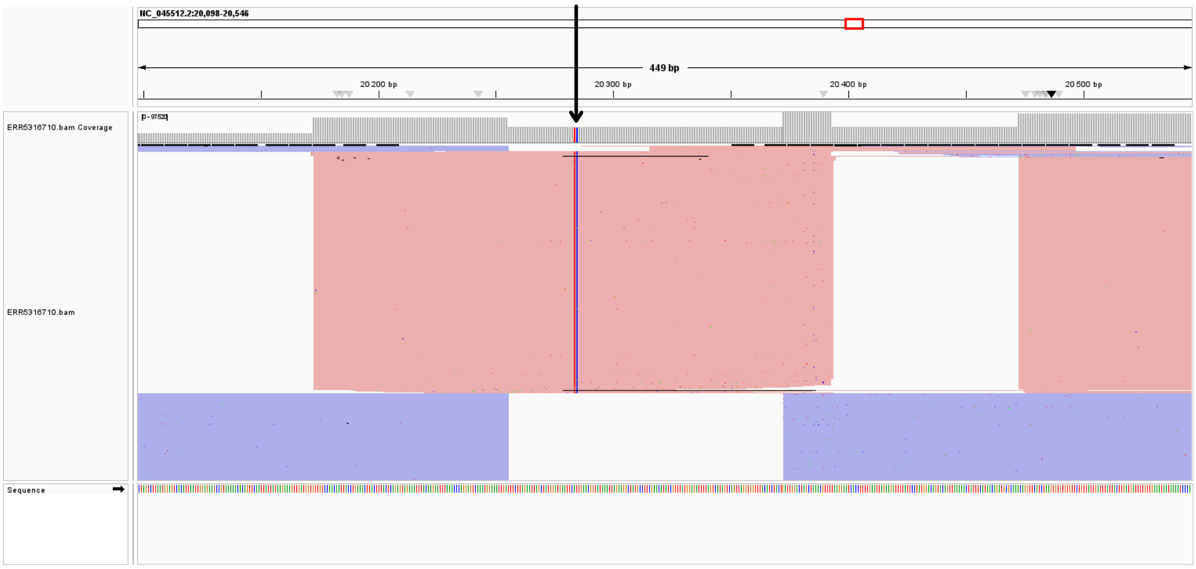

Sample ERR6930561: Nothing odd noted.

References

[1] Li, H. (2018). Minimap2: pairwise alignment for nucleotide sequences. Bioinformatics, 34:3094-3100. doi:10.1093/bioinformatics/bty191

[2] Danecek, P., Bonfield, J. K., Liddle, J., Marshall, J., Ohan, V., Pollard, M. O., ... & Li, H. (2021). Twelve years of SAMtools and BCFtools. Gigascience, 10(2), giab008.

[3] Hunt, M., Hinrichs, A. S., Anderson, D., Karim, L., Dearlove, B. L., Knaggs, J., ... & IMSSC2 Laboratory Network Consortium. (2024). Addressing pandemic-wide systematic errors in the SARS-CoV-2 phylogeny. BioRxiv.

[4] Thorvaldsdóttir, H., Robinson, J. T., & Mesirov, J. P. (2013). Integrative Genomics Viewer (IGV): high-performance genomics data visualization and exploration. Briefings in bioinformatics, 14(2), 178-192.

[5] Katoh, K., & Toh, H. (2008). Recent developments in the MAFFT multiple sequence alignment program. Briefings in bioinformatics, 9(4), 286-298.
