# Supplementary material for: Highly Recurrent Multinucleotide Mutations in SARS-CoV-2
Source: Mol Biol Evol. 2025 Oct 24;42(11):msaf272. doi: 10.1093/molbev/msaf272 (PMC12619124; doi:10.1093/molbev/msaf272)
Supplement: msaf272_Supplementary_Data [file msaf272_supplementary_data.zip › Supplement References.docx]

**Supplement References**

Katoh, Kazutaka et al. (2002). “MAFFT: a novel method for rapid multiple sequence alignment based on fast Fourier transform”. In: *Nucleic Acids Research* 30.14, pp. 3059–3066.

Krzywinski, Martin and Naomi Altman (2014). “Comparing samples—part II”. In: *Nature Methods* 11, pp. 355–357.
