# Supplementary material for: Highly Recurrent Multinucleotide Mutations in SARS-CoV-2
Source: Mol Biol Evol. 2025 Oct 24;42(11):msaf272. doi: 10.1093/molbev/msaf272 (PMC12619124; doi:10.1093/molbev/msaf272)
Supplement: msaf272_Supplementary_Data [file msaf272_supplementary_data.zip › supplement.pdf]

Supplementary Figures

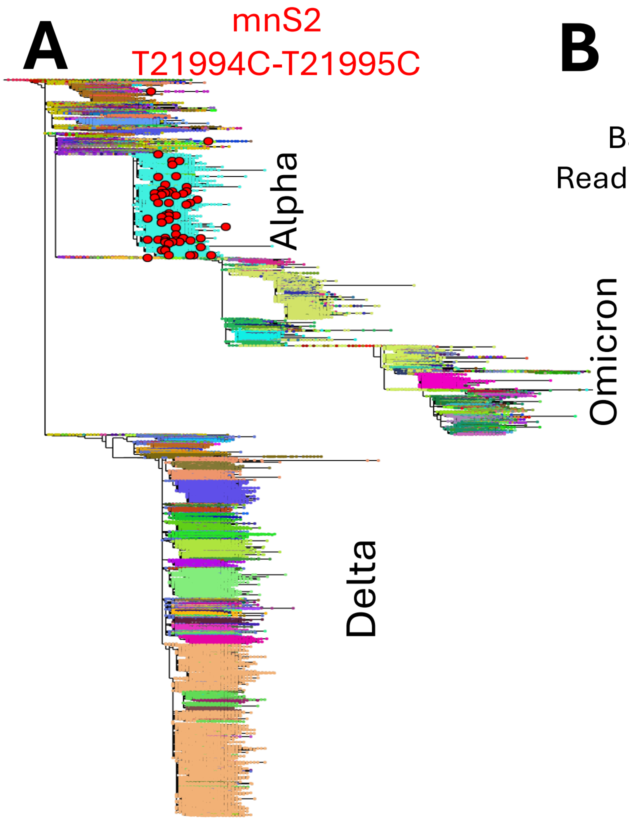

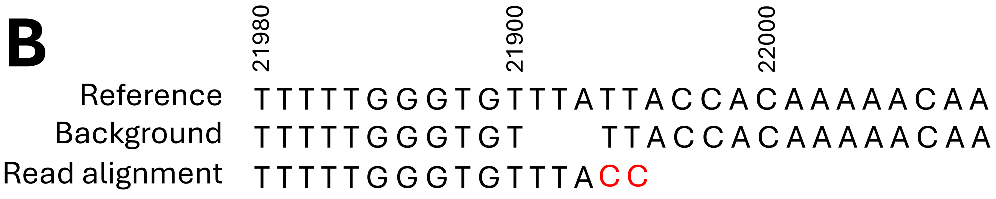

C

Position	Reference	Consensus	Depth
21988	T	T	88
21989	G	G	86
21990	T	T	80
21991	T	T	80
21992	T	T	80
21993	A	A	80
21994	T	C	64
21995	T	C	63
21996	A	A	58
21997	C	C	58
21997	-	N	6
21998	C	N	24
21999	A	N	24
22000	C	N	22
22001	A	N	22
22002	A	A	43
22003	A	A	46
22004	A	A	46
22005	A	A	47

Figure S1: **Issues with mnS2**. **A** SARS-COV-2 phylogenetic tree annotated (red circles) with occurrences of mnS2 (T21994C-T21995C). Details are as in Fig. 3. This mnS almost exclusively occurs in the Alpha lineage, where a deletion at positions 21991–21993 is ancestral to most genomes. **B** Local reference genome sequence ("Reference"), the background genome in Alpha ("Background", showing the deletion at positions 21991–21993 prevalent in the Alpha variant), and an example of a read alignment observed in samples containing mnS2 ("Read alignment"). The mis-aligned nucleotides causing the T21994C-T21995C mnS in the consensus genome are highlighted in red. These are typically found at the 3′ end of the read, where read alignment can favor two substitutions over a 3-nt deletion. **C** Viridian[36] quality control data for a random sample (ERR6719906) among the 28 containing a singleton T21994C-T21995C mnS (the mnS is inferred to have occurred on the terminal branch connecting the sample to the rest of the tree) and that are part of a phylogenetic cherry. We show reference position, reference nucleotide, Viridian consensus nucleotide, and total depth (a subset of the fields shown in Supplementary Table S1). Read alignment errors shift read depth to the deleted positions 21991–21993, making them appear not deleted in the consensus sequence, causing the T21994C-T21995C mnS, and leaving insufficient depth at positions 21998–22001 to call the reference genome. While this represents only one example genome, Supplementary Table S2 shows that 26/28 considered samples descending from a singleton T21994C-T21995C mnS and part of a cherry present low depth positions near the mnS, and visual inspection confirms that most of them have a similar pattern in read data alignment to that shown here.

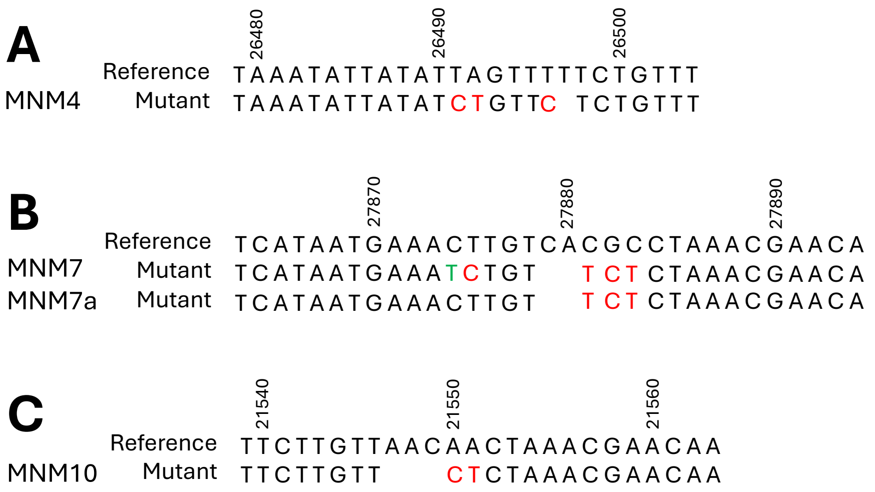

Figure S2: **Complex MNMs**. Some recurrent MNMs appear to involve deletions as well as substitutions. We show the reference genome sequence ("Reference") near the affected MNMs, as well as the sequence mutated by the MNM ("Mutant"). Nucleotide substitutions defining the MNM are marked in red. **A** MNM4 (T26491C-A26492T-T26497C) involves a 1-nt deletion. **B** MNM7 (T27875C-C27881T-G27882C-C27883T) and MNM7a (C27881T-G27882C-C27883T) involve 2-nt deletions; the green T in the MNM7 mutated sequence is part of the genomic background in most phylogenetic locations where MNM7 occurs (see Fig. 3). **C** MNM10 (A21550C-A21551T) involves a 3-nt deletion.

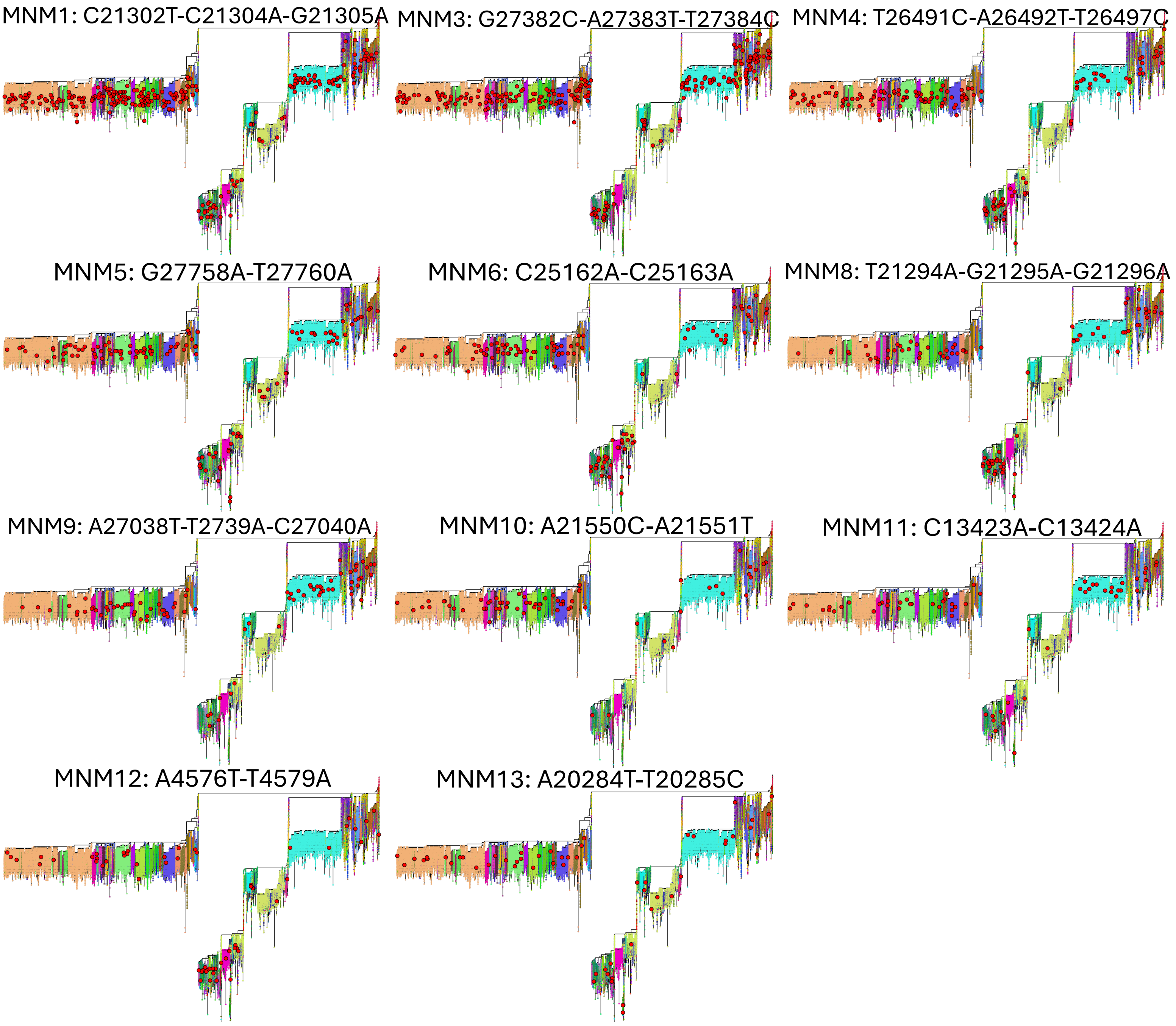

Figure S3: **Phylogenetic distributions of recurrent mnSs**. Each copy of the SARS-CoV-2 phylogenetic tree (details as in Fig. 3) is annotated (red circles) with occurrences of a different recurrent mnS. These mnSs do not show obvious phylogenetic clustering.

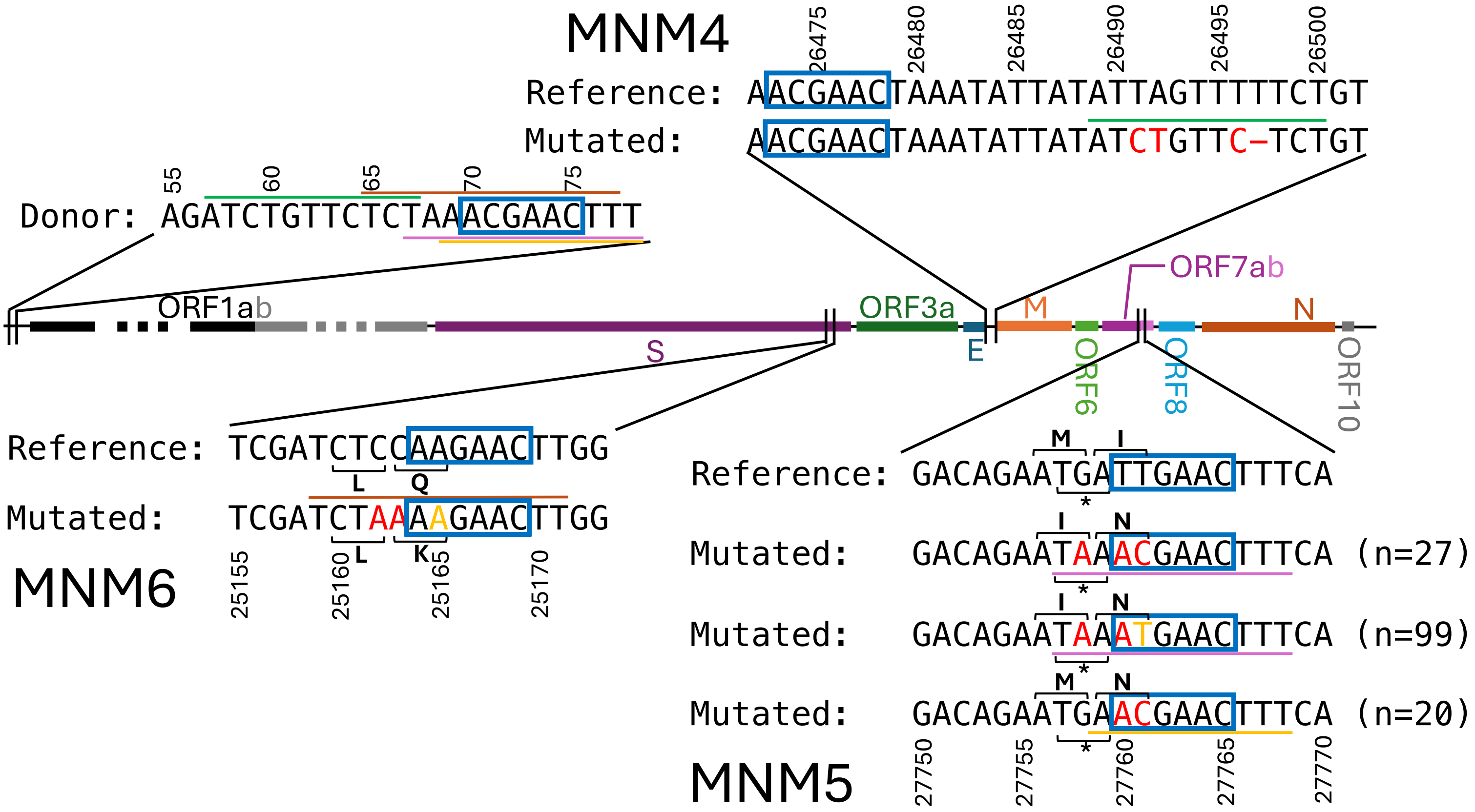

Figure S4: **Suggested origin of MNM4, MNM5, and MNM6**. Details are as in Fig. 6. Orange nucleotides are mismatches with respect to the highlighted TRS-L region of identity. At bottom right, we show MNM5 (G27758A-T27760A) together with its extended version G27758A-T27760A-T27761C (27 occurrences out of all 126 occurrences of MNM5) and its alternative T27760A-T27761C (20 occurrences) which are not recurrent enough to be listed in Table 1 on their own. These sequences are annotated with their translations as the end of ORF7a (below sequence) and the start of ORF7b (above). The M1I amino acid substitution caused by G27758A appears likely to knock out ORF7b.

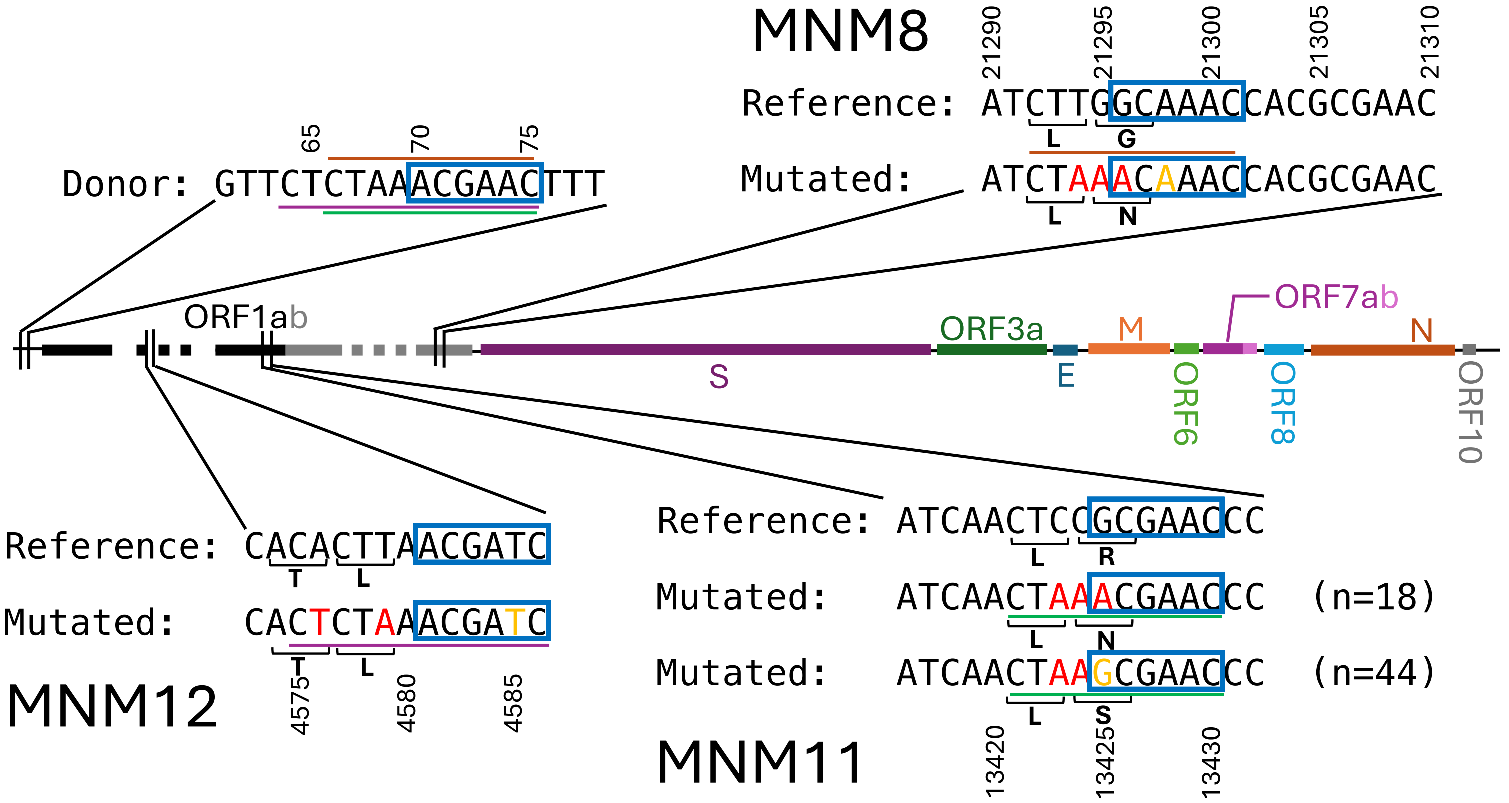

Figure S5: **Suggested origin of MNM8, MNM11 and MNM12**. Details are as in Fig. 6. Orange nucleotides are mismatches with respect to the highlighted TRS-L region of identity. We show two versions of MNM11 (C13423A-C13424A), one of which includes also substitution G13425A and which is not recurrent enough (18 occurrences out of all 62 occurrences of the cluster) to be listed in Table 1 on its own.

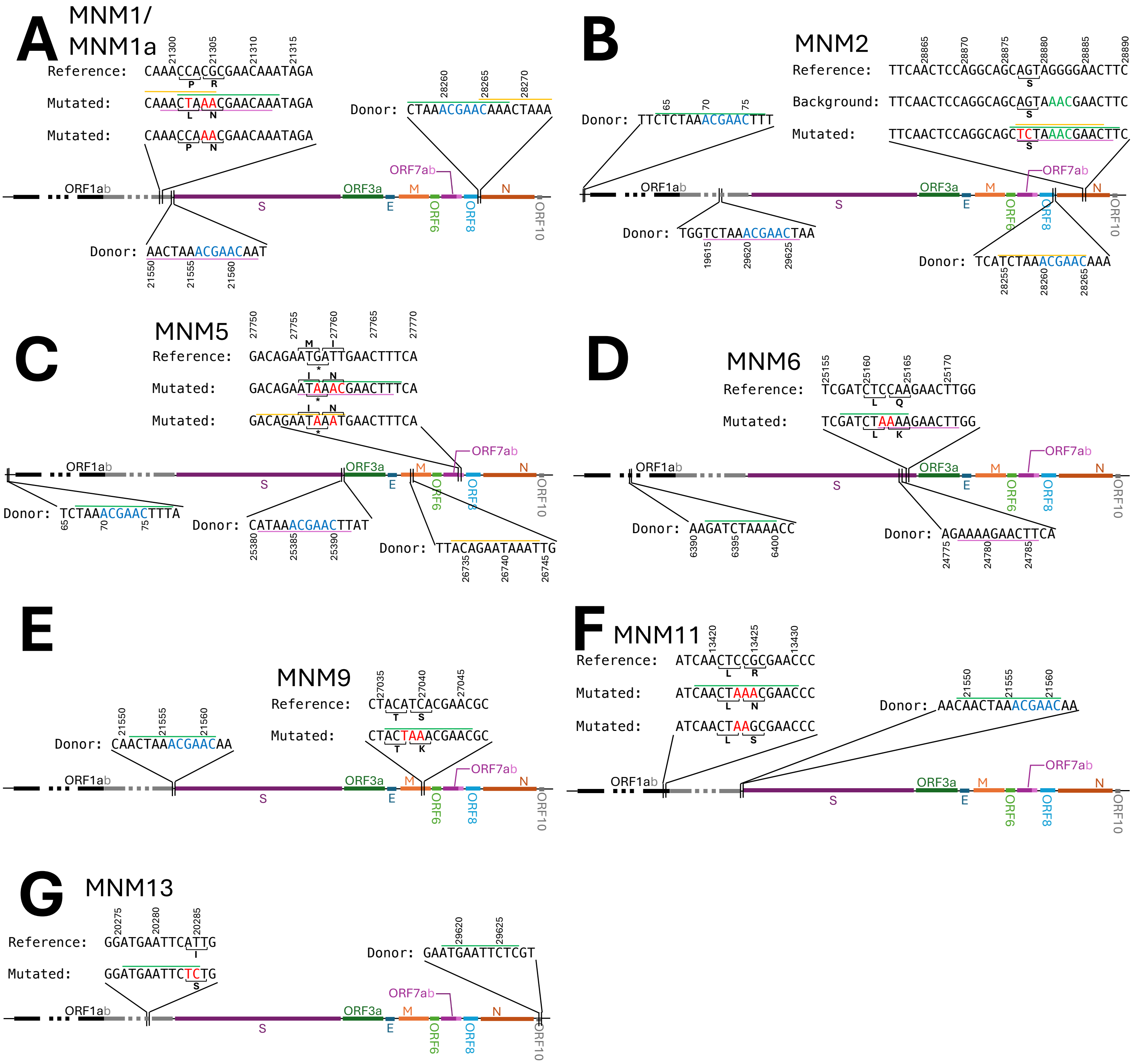

Figure S6: **Possible other templates in the SARS-CoV-2 genome**. Here we show regions of the SARS-CoV-2 genome, distinct from the TRS-L, that have long sequence identity (⩾9bp) with the mutated version of some of the recurrent MNMs. Details are as in Fig. 6. We highlight in blue the TRS consensus motif ACGAAC found in many of these regions. **A** MNM1 (C21302T-C21304A-G21305A) and MNM1a (C21304A-G21305A) (see also Fig. 6). **B** MNM2 (A28877T-G28878C) (see also Fig. 6). **C** MNM5 (G27758A-T27760A) (see also Supplementary Fig. S4). **D** MNM6 (C25162A-C25163A) (see also Supplementary Fig. S4). **E** MNM9 (A27038T-T27039A-C27040A) (see also Fig. 7). **F** MNM11 (C13423A-C13424A) (see also Supplementary Fig. S5). **G** MNM13 (A20284T-T20285C), the only recurrent MNM we could not explain through interrupted template switching.

Supplementary Tables

Position	Reference	Consensus	Depth	Clean depth	Consensus clean depth	Amplicons	Primers
497	A	A	1789	1279	1275	r1_1.1.409836; r1_1.1.592753; r1_1.1.876634	r1_1.1.876634_r_0; r1_1.1.409836_l_0
498	C	C	1789	1279	1271	r1_1.1.409836; r1_1.1.592753; r1_1.1.876634	r1_1.1.876634_r_0; r1_1.1.409836_l_0
499	T	T	1787	1278	1278	r1_1.1.409836; r1_1.1.592753; r1_1.1.876634	r1_1.1.876634_r_0; r1_1.1.409836_l_0
500	G	G	1787	1278	1276	r1_1.1.409836; r1_1.1.592753; r1_1.1.876634	r1_1.1.876634_r_0; r1_1.1.409836_l_0
501	C	C	1786	1277	1277	r1_1.1.409836; r1_1.1.592753; r1_1.1.876634	r1_1.1.876634_r_0; r1_1.1.409836_l_0
502	A	A	1785	1276	1275	r1_1.1.409836; r1_1.1.592753; r1_1.1.876634	r1_1.1.876634_r_0; r1_1.1.409836_l_0
503	C	C	1535	1028	1027	r1_1.1.409836; r1_1.1.592753; r1_1.1.876634	r1_1.1.876634_r_0; r1_1.1.409836_l_0
504	C	C	1751	1253	1004	r1_1.1.409836; r1_1.1.592753; r1_1.1.876634	r1_1.1.876634_r_0; r1_1.1.409836_l_0
505	T	T	1746	1249	999	r1_1.1.409836; r1_1.1.592753; r1_1.1.876634	r1_1.1.876634_r_0; r1_1.1.409836_l_0
506	C	C	1740	1246	1244	r1_1.1.409836; r1_1.1.592753; r1_1.1.876634	r1_1.1.876634_r_0; r1_1.1.409836_l_0
507	A	-	.	.	.	r1_1.1.409836; r1_1.1.592753; r1_1.1.876634	r1_1.1.876634_r_0; r1_1.1.409836_l_0
508	T	-	.	.	.	r1_1.1.409836; r1_1.1.592753; r1_1.1.876634	r1_1.1.876634_r_0; r1_1.1.409836_l_0
509	G	-	.	.	.	r1_1.1.409836; r1_1.1.592753	r1_1.1.409836_l_0
510	G	-	.	.	.	r1_1.1.409836; r1_1.1.592753	r1_1.1.409836_l_0
511	T	T	255	255	252	r1_1.1.409836; r1_1.1.592753	r1_1.1.409836_l_0
512	C	C	256	256	253	r1_1.1.409836; r1_1.1.592753	r1_1.1.409836_l_0
513	A	A	255	255	252	r1_1.1.409836; r1_1.1.592753	r1_1.1.409836_l_0
514	T	-	.	.	.	r1_1.1.409836; r1_1.1.592753	.
515	G	-	.	.	.	r1_1.1.409836; r1_1.1.592753	.
516	T	-	.	.	.	r1_1.1.409836; r1_1.1.592753	.
517	T	-	.	.	.	r1_1.1.409836; r1_1.1.592753	.
518	A	-	.	.	.	r1_1.1.409836; r1_1.1.592753	.
519	T	-	.	.	.	r1_1.1.409836; r1_1.1.592753	.
520	G	-	.	.	.	r1_1.1.409836; r1_1.1.592753	.
521	G	-	.	.	.	r1_1.1.409836; r1_1.1.592753	.
522	T	-	.	.	.	r1_1.1.409836; r1_1.1.592753	.
523	T	T	253	253	252	r1_1.1.409836; r1_1.1.592753	.
524	G	G	253	253	249	r1_1.1.409836; r1_1.1.592753	.
525	A	A	253	253	252	r1_1.1.409836; r1_1.1.592753	.
526	G	G	251	251	243	r1_1.1.409836; r1_1.1.592753	.
527	C	C	253	253	250	r1_1.1.409836; r1_1.1.592753	.
528	T	T	253	253	250	r1_1.1.409836; r1_1.1.592753	.
529	G	G	253	253	253	r1_1.1.409836; r1_1.1.592753	.
530	G	G	253	253	253	r1_1.1.409836; r1_1.1.592753	.
531	T	T	253	253	252	r1_1.1.409836; r1_1.1.592753	.
532	A	A	253	253	253	r1_1.1.409836; r1_1.1.592753	.
533	G	G	253	253	253	r1_1.1.409836; r1_1.1.592753	.
534	C	C	253	253	253	r1_1.1.409836; r1_1.1.592753	.
535	A	A	252	252	252	r1_1.1.409836; r1_1.1.592753	.
536	G	N	252	0	0	r1_1.1.409836; r1_1.1.592753	r1_1.1.592753_r_0
537	A	N	245	0	0	r1_1.1.409836; r1_1.1.592753	r1_1.1.592753_r_0
538	A	N	244	0	0	r1_1.1.409836; r1_1.1.592753	r1_1.1.592753_r_0
539	C	N	245	0	0	r1_1.1.409836; r1_1.1.592753	r1_1.1.592753_r_0
540	T	N	245	0	0	r1_1.1.409836; r1_1.1.592753	r1_1.1.592753_r_0
541	C	N	245	0	0	r1_1.1.409836; r1_1.1.592753	r1_1.1.592753_r_0
542	G	N	245	0	0	r1_1.1.409836; r1_1.1.592753	r1_1.1.592753_r_0
543	A	N	245	0	0	r1_1.1.409836; r1_1.1.592753	r1_1.1.592753_r_0
544	A	N	245	0	0	r1_1.1.409836; r1_1.1.592753	r1_1.1.592753_r_0
545	G	N	244	0	0	r1_1.1.409836; r1_1.1.592753	r1_1.1.592753_r_0

Table S1: Issues in sequencing read data for sample SRR19943422. We show the Viridian[36] quality control data for a random sample among the 32 containing a singleton mnS1 (A507T-T508C-G509A) substitution and that are part of a phylogenetic cherry. We show only data for positions near the considered mnS.
Position: reference genome position. Reference: reference nucleotide at the position. Consensus: consensus nucleotide called by Viridian from the read data at the considered position for the considered sample. Depth: sequencing read depth at the position. Clean depth: depth after trimming primers from the reads. Consensus clean depth: clean depth supporting the Viridian consensus nucleotide at the position. Amplicons: amplicons covering the position for the sequencing protocol inferred by Viridian. Primers: primers covering the position for the sequencing scheme inferred by Viridian.
The table shows that following a region of high depth up to reference position 506, depth suddenly drops, and read alignments contain deletions (unlike the sibling genomes in the phylogenetic cherry, not shown). Also, within-sample heterozygosity is observed at positions 504 and 505 (the consensus clean depth is substantially lower than the total clean depth). After position 536 the consensus sequence is masked by Viridian (the consensus nucleotide is N) due to primer r1_1.1.592753_r_0, and apparently due to dropout of amplicon r1_1.1.409836. All these observations suggest that the herozygosity and indels might be due to inaccurate primer identification and consequentially incomplete primer trimming. Aligning the consensus Viridian genome to the reference with MAFFT[72] (see details in [35]) introduces substitutions A507T, T508C, and G509A, and a deletion. While this table highlights just one example, Supplementary Table S2 shows that all considered samples descending from a singleton mnS1 substitution and part of a cherry also present heterozygosity and indels at these positions, and visual inspection of their read data further led us to conclude that this recurrent mnS is the consequence of sequencing artefacts.

ID	mnS	Phylogenetic clustering	Reversions	Deletions in cherries	Low coverage in cherries	Heterozygosities in cherries
MNM1	C21302T -C21304A -G21305A		0	0/78	5/78	12/78
> MNM1a	C21304A -G21305A		0	0/97	4/97	6/97
MNM2	A28877T -G28878C	✓	13	1/131	2/131	13/131
MNM3	G27382C -A27383T -T27384C		5	0/69	1/69	5/69
MNM4	T26491C -A26492T -T26497C		0	51/51	0/51	7/51
MNM5	G27758A -T27760A		0	0/37	1/37	6/37
MNM6	C25162A -C25163A		0	0/29	0/29	3/29
MNM7	T27875C -C27881T -G27882C -C27883T	✓	0	9/9	0/9	0/9
> MNM7a	C27881T -G27882C -C27883T		0	23/23	0/23	4/23
MNM8	T21294A -G21295A -G21296A		0	0/21	1/21	0/21
mnS1	A507T -T508C -G509A		0	32/32	2/32	32/32
MNM9	A27038T -T27039A -C27040A		0	0/26	3/26	6/26
MNM10	A21550C -A21551T		0	16/16	0/16	3/16
mnS2	T21994C -T21995C	✓	2	7/28	26/28	0/28
MNM11	C13423A -C13424A		0	0/24	0/24	0/24
MNM12	A4576T -T4579A		0	0/14	4/14	0/14
mnS3	T28881A -G28882A -G28883C		9	12/16	0/16	3/16
MNM13	A20284T -T20285C		0	0/14	0/14	0/14
mnS4	G11083T -C21575T		0	0/14	0/14	14/14

Table S2: Other features of highly recurrent mnSs. mnSs unlikely to be caused by recurrent MNMs are highlighted in red. Phylogenetic clustering: mnSs clearly non-homogeneously spread along the phylogenetic tree (as in Supplementary Fig. S3) are marked with ✓. Reversions: Number of flipped substitutions inferred for the considered mnS; e.g. A21304C-A21305G is the reversion of MNM1a (C21304A-G21305A). The last three columns list how many of the investigated samples containing singletons for the considered mnS also contain near the same mnS: deletions in the consensus sequence; low (<100) read coverage positions; and heterozygosities (>5% non-consensus nucleotide frequency in the read alignment). We consider positions $\leqslant$20bp before the first base in the mnS, or $\leqslant$25bp after it. For example, for the first row, out of 78 samples containing a singleton MNM1 mutation (C21302T-C21304A-G21305A) and with a sibling sample in the tree, 5 contained low read coverage in at least one site between genome positions 21282 and 21327.

mnS	Number of occurrences	Individual occurrences	p-value	Occurrences in simulations
C21302T -C21304A	52	503 1105	$9 \cdot 10^{-116}$ $(7 \cdot 10^{-106})$	0 (0–3)
G27382C -A27383T	46	388 328	10^{-130} $(8 \cdot 10^{-121})$	0 (0–1)
C22716A -T22717C	44	48 52	$5 \cdot 10^{-230}$ $(4 \cdot 10^{-220})$	0 (0–0)
T27672A -C27673A	37	47 179	$3 \cdot 10^{-154}$ $(2 \cdot 10^{-144})$	0 (0–1)
G910A -T911A -C912A	36	75 37 84	$3 \cdot 10^{-174}$ $(2 \cdot 10^{-164})$	0 (0–0)
G6975T -G6977A	33	46 62	$2 \cdot 10^{-153}$ (10^{-143})	0 (0–1)
G6513A -T6515A	33	46 34	10^{-169} (10^{-159})	0 (0–1)
T27381C -G27382T -A27383G	32	61 217 70	$9 \cdot 10^{-140}$ $(7 \cdot 10^{-130})$	0 (0–0)
C19977A -C19979A	32	40 57	$4 \cdot 10^{-153}$ $(3 \cdot 10^{-153})$	0 (0–0)
T22207G -T22209C	31	53 43	$4 \cdot 10^{-147}$ $(3 \cdot 10^{-137})$	0 (0–0)
A27865T -T27866A	29	41 33	$4 \cdot 10^{-147}$ $(3 \cdot 10^{-137})$	0 (0–0)
A21892G -G21893A	28	44 72	$5 \cdot 10^{-125}$ $(4 \cdot 10^{-115})$	0 (0–1)
A5703T -G5704A -T5705A	27	36 58 28	$6 \cdot 10^{-142}$ $(6 \cdot 10^{-106})$	0 (0–0)
T26485C -T26486A	27	45 31	10^{-134} (10^{-124})	0 (0–0)
G11071C -C11074T	26	79 1355	10^{-70} $(8 \cdot 10^{-61})$	0 (0–1)
C17734T -T17735C	25	94 26	$6 \cdot 10^{-117}$ $(5 \cdot 10^{-107})$	0 (0–1)
T27299C -A27300G	24	140 27	$2 \cdot 10^{-105}$ (10^{-95})	0 (0–1)
A3684T -G3685A -C3686A	23	29 74 83	$6 \cdot 10^{-106}$ $(5 \cdot 10^{-96})$	0 (0–0)
C21302T -T21304A -G21305A	23	503 41 1026	10^{-79} $(8 \cdot 10^{-70})$	0 (0–0)
G23957T -T23959G	20	22 23	$4 \cdot 10^{-109}$ $(4 \cdot 10^{-99})$	0 (0–0)
A25562T -G25563A	20	43 51	$8 \cdot 10^{-89}$ $(7 \cdot 10^{-79})$	0 (0–0)
T27760A -T27761C	20	186 61	$2 \cdot 10^{-72}$ $(2 \cdot 10^{-62})$	0 (0–0)

Table S3: List of mnSs with at least 20 occurrences, not included in Table 1, and unlikely to be due to recurrent individual nucleotide mutations. Columns refer to the same values as in Table 1. The number of individual occurrences includes also occurrences as part of the considered mnS (so numbers of "Individual occurrences" are necessarily higher than the "Number of occurrences"). These mnSs have not been investigated in detail, so we do not exclude that they might be attributable to sequencing issues rather than MNMs.

mnS	Number of occurrences	Individual occurrences	p-value	Occurrences in simulations
C11074T<br>-G11083T	41	1355<br>11259	$8 \cdot 10^{-29}$<br>$(6 \cdot 10^{-19})$	13 (4–22)
A10323G<br>-C21575T	27	2852<br>6966	$2 \cdot 10^{-12}$<br>(0.02)	16 (8–26)
G11083T<br>-C29095T	24	11259<br>1673	10^{-10}<br>(0.90)	16 (8–30)
G11083T<br>-C16887T	23	11259<br>3280	$6 \cdot 10^{-5}$<br>1	31 (21–52)
G11083T<br>-C29614T	21	11259<br>1830	$7 \cdot 10^{-8}$<br>1	17 (6–26)
G11083T<br>-T27384C	21	11259<br>1977	10^{-6}<br>1	19 (9–28)
G11083T<br>-C26681T	20	11259<br>2096	$2 \cdot 10^{-6}$<br>1	19 (9–28)
G1820A<br>-G11083T	20	2146<br>11259	$3 \cdot 10^{-6}$<br>1	19 (9–32)
G11083T<br>-A21137G	20	11259<br>2661	0.0002<br>(> 1)	25 (13–37)

Table S4: List of mnSs with at least 20 occurrences, not included in Table 1, and likely to be due to recurrent individual nucleotide mutations. Columns refer to the same values as in Table 1. The number of individual occurrences includes also occurrences as part of the considered mnS (so numbers of "Individual occurrences" are necessarily higher than the "Number of occurrences"). Bonferroni-corrected p-values are capped at 1[73].
